# Enhancing the European power system resilience with a recommendation system for voluntary demand response

**DOI:** 10.1016/j.isci.2024.111430

**Published:** 2024-11-19

**Authors:** Carlos A.M. Silva, Ricardo J. Bessa, José R. Andrade, Fábio A. Coelho, Rafael B. Costa, Carlos Damas Silva, George Vlachodimitropoulos, Donatos Stavropoulos, Spiros Chadoulos, David E. Rua

**Affiliations:** 1 INESC TEC, Center for Power and Energy Systems, Porto, Portugal; 2 Faculty of Engineering, University of Porto, Porto, Portugal; 3 INESC TEC & University of Minho, High-Assurance Software Laboratory, Braga, Portugal; 4 E-REDES, Lisbon, Portugal; 5 University of Aegean, Samos, Greece; 6 Local AI, Kalamata, Greece; 7 GRIDNET, Volos, Greece; 8 Mobile Multimedia Laboratory, Department of Informatics, School of Information Sciences and Technology, Athens University of Economics and Business, Athens, Greece

**Keywords:** Engineering, Energy engineering, Energy systems

## Abstract

Climate change, geopolitical tensions, and decarbonization targets are bringing the resilience of the European electric power system to the forefront of discussion. Among various regulatory and technological solutions, voluntary demand response can help balance generation and demand during periods of energy scarcity or renewable energy generation surplus. This work presents an open data service called Interoperable Recommender that leverages publicly accessible data to calculate a country-specific operational balancing risk, providing actionable recommendations to empower citizens toward adaptive energy consumption, considering interconnections and local grid constraints. Using semantic interoperability, it enables third-party services to enhance energy management and customize applications to consumers. Real-world pilots in Portugal, Greece, and Croatia with over 300 consumers demonstrated the effectiveness of providing signals across diverse contexts. For instance, in Portugal, 7% of the hours included actionable recommendations, and metering data revealed a consumption decrease of 4% during periods when consumers were requested to lower consumption.

## Highlights

• Interoperable Recommender offers load increase or decrease recommendations to consumers
• Service based on open data to calculate country-specific operational balancing risk
• Pilot projects demonstrate potential for voluntary demand response

## Graphical abstract

**Figure:**
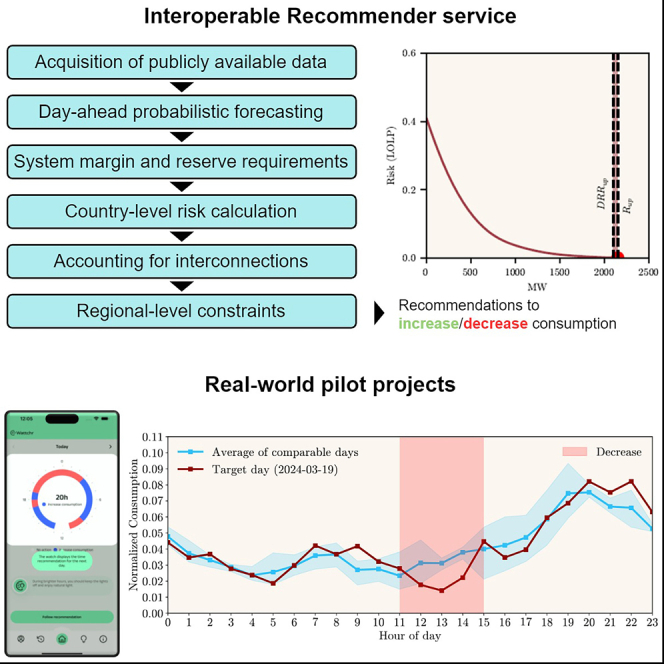

## Introduction

Society and the economy rely on the bulk power system as a critical infrastructure to ensure a continuous, reliable, and efficient electricity supply. Any disruption in its normal operation can significantly affect individuals, have meaningful social and economic effects, and even cascade to other critical systems.^1^ The electrical grid is crucial for national economies and economic growth, as investments in grid development are directly linked to economic prosperity.^2^ However, as we witness the electrification of various sectors^3^ and the increasing installed capacity of renewable energy sources (RES), concerns about the vulnerability of the electrical power system have become more noticeable.^4^^,^^5^ Whether during normal operation or a disruptive event, there is a need for a resilient power system, often taken for granted.

Europe is facing a challenging and uncertain environment. Recent events, such as the invasion of Ukraine, have highlighted the vulnerability of the energy system, with energy resources such as natural gas supply being jeopardized.^6^ This has contributed to an energy crisis in Europe, reducing the availability of natural gas resources and creating challenges for citizens and businesses in managing their energy bills. Additionally, the impact of climate change is a growing concern,^7^ leading to more frequent, intense, and prolonged extreme weather events that stress the transmission infrastructure and can cause system blackouts.^8^ An example from the United States was the winter storms in Texas, which resulted in widespread generation capacity outages with critical effects.^9^

The large-scale deployment of RES is critical for decarbonizing electricity generation, enhancing energy supply security, and decreasing the dependency on geopolitical interests. Still, the inherent variability of RES and the forecast uncertainty pose challenges for planning and operating the electrical power system.^10^ Hence, ensuring a resilient power system is essential in the path toward a sustainable energy system,^11^ and it should be a policy priority at both the European and national levels.^6^^,^^12^ Strategies such as network infrastructure upgrades, demand-side flexibility, and deployment of smart grid technologies are crucial to effectively monitor and manage the electric power system.^13^

Demand-side flexibility, in particular, plays a significant role in adapting the energy use to the operational conditions, even during disruptive events.^14^ It encompasses price or incentive-based programs, as well as non-monetary approaches, aimed at encouraging consumers to adjust their energy consumption patterns.^15^ Recent research has shown the importance of exploring non-economic incentives to promote demand response (DR), as evidenced by field experiments conducted by Shadid et al.,^16^ Scharnhorst et al.,^17^ and Pratt and Erickson.^18^ It is important to note that the concept of appealing to consumers for energy conservation is not new and can be traced back to the oil crisis in the 1970s.^19^

Nowadays, these strategies rely heavily on the digitalization of the energy system,^20^ including smart devices, internet-of-things (IoT), and innovative digital platforms for consumers to manage their energy use. Following recent reports published by the European Commission (EC),^21^^,^^22^ and the Smart Grids Task Force,^23^ the European H2020 Project InterConnect contributed to demonstrate, in 12 countries, the first generation of the blueprint for a Common European Reference Framework (CERF) for energy-saving applications. The CERF emphasizes the importance of semantic interoperability for seamless information exchange between services and data sources.^24^ The InterConnect Semantic Interoperability Framework (SIF), based on the Smart Applications REFerence (SAREF) ontology,^25^ provides the set of tools that enable energy stakeholders to communicate in an interoperable manner (achieved at the software stack level) with third parties that use data-driven energy and non-energy digital services. Being semantically interoperable means that digital services exchange payloads of information in which the meaning of data, its context, and units of measurement are detailed through an unambiguous representation (i.e., an ontology), unlocking the capability to interpret data and apply translations if required (e.g., on the fly translations of units of measurement). These accomplishments drove the development of an ecosystem of collaborative demand-side management strategies at the European level, empowering consumers to adapt their energy use. Indeed, the collaboration of civil society, namely energy consumers, can be a key resource for innovation within the energy transition context.^26^

This article describes the methods and results from the InterConnect project’s open data-driven service called Interoperable Recommender (IR), further detailed in the STAR Methods section, which leverages open data platforms such as the ENTSO-E Transparency Platform^27^ and semantic interoperability to produce recommendations to be passed to consumers via energy-saving mobile applications. The IR determines a set of nationally day-ahead targeted actions to increase or decrease energy consumption during specific periods to contribute to the resilience of the European electricity system, particularly focused on load-generation balancing at the national level. The IR has been operational since August 2023, providing valuable insights to several EU member states. Given that demand-side management strategies need regional, national, and European coordination, the developed methodology employs a hierarchical approach. This approach simultaneously considers European cross-border exchanges, country-level power system imbalances, and local operational constraints (e.g., at the local utility level) of the electricity distribution networks to minimize operational balancing risk. Here, the risk is represented by the probability of failing to satisfy the load or curtail RES surplus in the short term (e.g., as the result of forecast errors) to keep generation and load balanced. It is important to note that this hierarchical approach seeks to promote close cooperation and exchange of information between system operators at different levels (e.g., cross-country, national, and regional) when DR resources connected to distribution grids (utility level) are used for system balancing. Thus, this real-life pilot contributes toward more advanced transmission-distribution systems coordination schemes,^28^ where the system balancing task has to co-exist with utility-level tasks such as congestion and voltage management.

Related real-world implementations further illustrate the effectiveness of demand-side management initiatives. For instance, EcoWatt^29^ operates as a platform in France that delivers signals to consumers through a traffic-light interface, signaling actions for decreasing or increasing consumption based on power system carbon intensity and system margins. Moreover, EcoWatt has an application programming interface (API) for developers to retrieve the signal and modify the programming of equipment or Building Management Systems in commercial buildings.^30^ Similarly, Flex Alert, managed by the Californian Independent System Operator, issues calls to consumers a day in advance, urging them to reduce energy consumption voluntarily during anticipated electricity supply shortages. Recent work by Peplinski and Sanders^31^ underscores both the benefits and limitations of this initiative. The Electricity Maps platform^32^ provides information related to country-level electricity mix and carbon intensity, which can be integrated into carbon-aware demand optimization.^33^ Another source that provides grid-related data such as the marginal and average operating emissions rate of carbon dioxide is the WattTime^34^ platform.

While the regulatory framework for DR at the European level is still evolving,^35^ exploring alternatives such as providing non-economic signals (i.e., alerts) directly to consumers on a voluntary basis presents a low-overhead solution. Many local experiments were developed for DR,^36^ but a research gap exists concerning delivering signals effectively at the European scale and communicating risk information (e.g., associated with energy scarcity or spilling renewable energy events) to citizens. Although many examples exist of using open data in the energy field, such as for forecasting,^37^ modeling realistic transmission networks,^38^ and building generator unavailability models for security of supply studies,^39^ the authors are not aware of recent work that uses open data sources to compute and provide country-level risk-based signals to consumers. Furthermore, the use of semantic interoperability is of increasing relevance in the energy field and demand response in particular,^40^^,^^41^ but large-scale implementations are still limited. To address these gaps, this work brings the following contributions: a) leveraging on open data to predict country-specific operational risk and derive actionable recommendations at the European level, considering interconnections and local network constraints, b) a service designed with interoperability in mind, facilitating seamless integration into third-party digital services, which enables a wide range of applications to benefit from the insights provided, and c) a showcase of results and conclusions from real-world pilots, using data collected during months, with mobile energy applications of diverse settings that use the IR as a software backbone. This work offers tangible, practical tools and insights that system operators and third-party services can readily adopt.

## Results

The results are organized into two sections. The first one analyzes the performance of the IR service for a period of 75 days, characterizing recommendations for multiple European countries that were part of the InterConnect project and detailing the results for Portugal as an example. The second offers a perspective of pilot projects in three different countries (Portugal, Greece, and Croatia) that used the IR service to promote voluntary DR with real consumers, including the specialization of the recommendations by the distribution system operator (DSO) to account for local network constraints.

### Setting country-level recommendations

This section introduces the basic operation of the IR service. As detailed in the IR service overview section, the service output is day-ahead hourly recommendations to increase or decrease the energy consumption for each country. More specifically, the recommendations (their direction and magnitude) are related to the requirements for the upward and downward reserves ($R_{up}$ and $R_{down}$) in each period, sized with a probabilistic approach that accounts for the system margin. Inspired by the well-being model for power systems,^42^ a deterministic rule for the reserve ($DRR_{up}$ and $DRR_{down}$) is used to classify the operating states as *healthy* or *at risk*. When the system deviates from *healthy* to *at risk* (i.e., the reserve requirements are greater than the deterministic rule), alerts are generated in the form of actionable recommendations for consumers. These alerts are computed for different European countries and provided to consumers to unlock demand flexibility on a voluntary basis. Moreover, the operation of this service and its associated alerts relies on open data provided by the ENTSO-E Transparency Platform and is consequently limited by the availability of this data. The results presented in this section relate to the outputs of the IR service between the 1^st^ of February and 15^*th*^ of April of 2024. The code related to the IR service can be found in the Data and code availability section.

**Figure 1:**
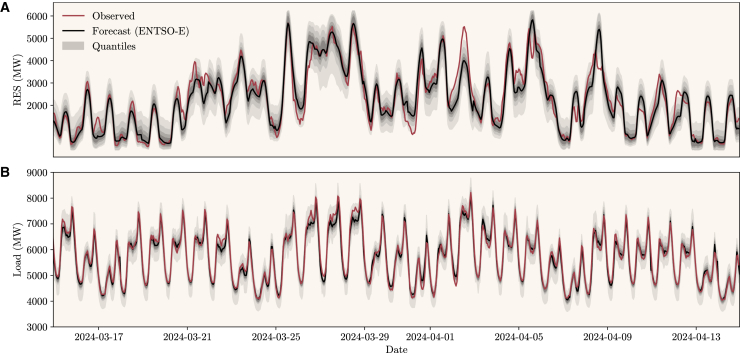
RES generation and Load forecasts, and observed values for Portugal (A and B) show, respectively, values for RES and load for Portugal (both in MW, between the $15^{th}$ of March and $15^{th}$ of April of 2024). Deterministic forecasts from ENTSO-E are depicted in black, and the observed values are in red. The shaded area (in gray) relates to the different predicted quantiles provided by the probabilistic forecasts using a linear quantile regression, which can capture the overall profile for the mentioned period.

First, we evaluate the quality of the RES and load probabilistic forecasts, a core input for the service. Details on the linear quantile regression employed for forecasting are available in the STAR Methods section. Thirty-one days of forecasted quantiles and observed values between March and April of 2024 are presented in Figure 1 for Portugal, where it is clear that the forecasted quantiles capture the overall observed patterns. A summary of probabilistic forecasting skill metrics, namely the Continuous Ranking Probability Score (CRPS) and the Interquantile Range (IQR) described in the STAR Methods Section and averaged for the whole testing period, is available in Table 1, where the countries included relate to the pilot projects developed within the Horizon 2020 InterConnect project. These metrics allow quantifying the forecasting skill of the probabilistic forecasts and their level of uncertainty (or sharpness). The IQR and CRPS metrics were normalized according to the total RES installed capacity of each country or its maximum load in the period considered. The table also includes the Mean Absolute Error (MAE) of the RES, Load, and Total Generation deterministic forecasts directly available from the ENTSO-E Transparency Platform, respectively normalized with each country’s RES installed capacity, maximum load observed during the mentioned period, and total system generation capacity.

**Table 1:** Deterministic and probabilistic forecasting metrics

Country	Normalized MAE(%)			Normalized IQR(%)		Normalized CRPS(%)	
	RES	Load	Total Gen.	RES	Load	RES	Load
BE	2.01	2.08	2.20	9.80	9.39	1.66	1.57
DE	3.67	2.24	0.79	11.71	10.31	2.93	1.43
DK	5.95	4.16	2.77	15.01	11.02	3.22	2.90
EE	3.60	4.87	1.76	14.92	21.97	2.66	3.65
ES	2.86	1.69	1.18	7.96	4.93	1.90	1.18
FR	4.34	2.09	2.67	9.84	7.90	3.10	1.45
GR	1.77	0.85	0.85	8.73	9.90	1.29	1.51
HR	3.88	3.34	2.68	18.42	10.29	2.87	1.80
IT	4.89	1.52	6.51	14.85	5.13	3.06	1.07
NL	3.19	8.64	5.86	9.97	21.50	1.97	6.07
PT	4.08	1.21	2.72	16.46	6.34	2.98	0.89
SI	0.56	2.62	4.69	2.75	12.26	0.44	1.88

More than half of the countries revealed a normalized MAE for RES above 3% (with Denmark reaching 6%) of the RES installed capacity. For the load, the MAE for all countries is below 4% (except for Denmark, Estonia, and the Netherlands, with the latter reaching over 8%) of the observed maximum load. For the total generation forecasts, the MAE is high for some countries (around 6% for Italy and the Netherlands) but is mostly under 3% of the total generation capacity. Regarding the probabilistic forecasts computed with the linear quantile regression, countries presented a $CRPS_{RES}$ mostly under 3% (with exceptions for Denmark, France, and Italy), and $CRPS_{Load}$ mostly under 2% (with an exception for Denmark, Estonia, and the Netherlands). The normalized $IQR_{RES}$ ranged between 3% and 19%, while the $IQR_{Load}$ ranged between 5% and 22%. In general, the deterministic forecasts performed better for the load than for the RES, which is expected due to the inherent variability of RES, such as wind and solar energy. This conclusion also extends to the CRPS metric computed for the probabilistic forecasts. The main findings, which reveal discrepancies between predicted and observed values for some countries, could be partially explained by issues related to inconsistencies and completeness, as reported previously by Hirth et al.^27^ for some variables available in the ENTSO-E Transparency Platform. Additional insights are reported by Kazmi and Tao,^43^ which analyze the performance of the day-ahead load and renewable energy forecasts available in the ENTSO-E Transparency Platform.

It is challenging to find literature reporting metrics on country-level load and RES generation probabilistic forecasts for comparison purposes. As an example for the load, Zimmermann and Ziel^44^ report a *CRPS* of 2% and 2.9% for Portugal and Spain (we normalized the reported values by each country’s maximum load of 2023 for a direct comparison), while 0.9% and 1.2% were obtained in this work. Still, the mentioned work considers a more extended period of analysis. As an example for RES forecasting, Andrade and Bessa^45^ report *CRPS* values of around 4% for a PV and a wind power forecasting model tested on specific locations in Portugal and Spain, respectively. Still, it is not directly comparable to the values obtained in the present document (3% and 1.9%, respectively), as we consider not a specific park but the country-level aggregated RES (PV + Wind) generation.

**Figure 2:**
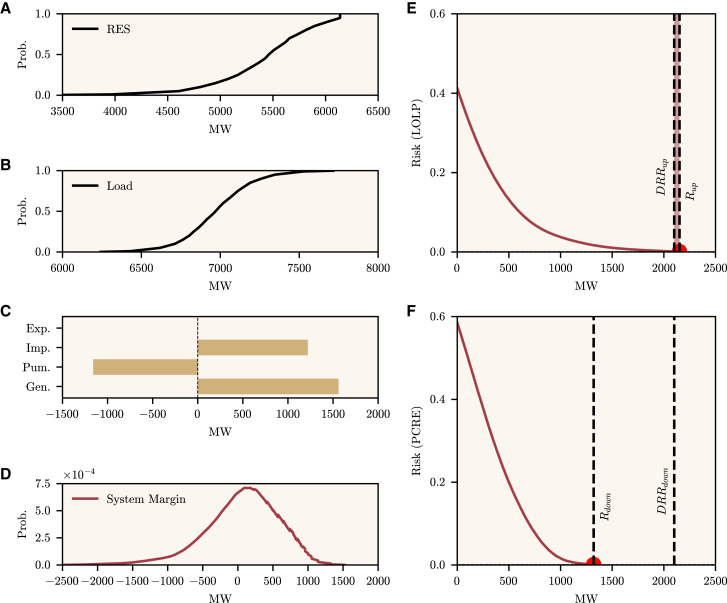
Calculation of reliability indices and reserve requirements (upward and downward) for a specific period All the values presented in the Figure are in MW and relate to data for a specific hour for Portugal to illustrate how the operating status of a country is determined (*healthy* or *at risk*). (A and B) show the Cumulative Distribution Function of RES and load, respectively. (C) depicts the deterministic information available related to scheduled commercial exchanges (Exports and Imports), Hydropower pumping, and Conventional Generation through a horizontal bar plot. (D) shows the computed System Margin through a convolution process in a red line, given the probabilistic and deterministic information provided from (A) to (C). (E and F) depict the risk-reserve curve, respectively, for the upward and downward reserve. In this period, the reserve requirements for the upward case are larger than the deterministic rule for the reserve (DRR), which means the country is considered *at risk* and an alert for consumers to decrease consumption is expected at this hour.

The provided recommendations are impacted by the uncertainty level of the day-ahead load and RES forecasts, influenced by the point forecast error magnitude. As indicated in the STAR Methods, the recommendations depend on the risk of loss of load or renewable energy curtailment. The more uncertain the forecasts, the larger the tails of the System Margin (Equations 3 and 4, as illustrated by Figure 2D). Consequently, the risk will increase since the operational reserve requirements will be higher (see Figure 2E as an example of a loss of load situation). The System State (Equation 9) ultimately computes what recommendation is provided and depends on the difference between the DRR and the reserve requirements. Therefore, higher forecast uncertainty can cause higher reserve requirements and, ultimately, change recommendations from *healthy* to *increase* or *decrease*. To assess how a larger uncertainty would impact recommendations, a sensitivity analysis would be needed, for example, by synthetically increasing or decreasing the amount of forecast uncertainty (or the forecast error magnitude). This analysis would depend on the country and season (e.g., the impact of the uncertainty is naturally lower in periods of lower RES generation or load).

**Figure 3:**
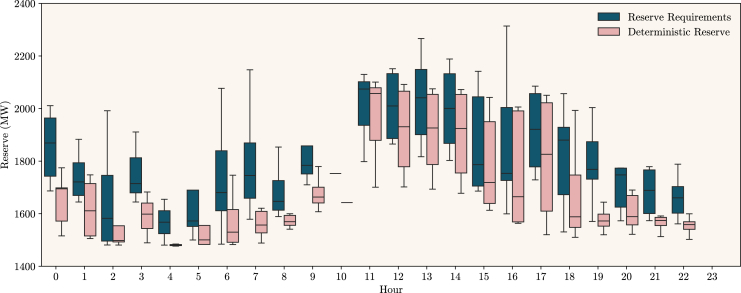
Reserve levels for Portugal A boxplot by hour of day shows the difference between the reserve requirements (in blue) and the deterministic rule for the reserve (in pink), both in MW, and for Portugal. This plot considers only hours where the country was *at risk* (i.e., the requirements were larger than the deterministic rule). Data for each hour consists of the first (Q1) and third (Q3) quartile, the median, and a lower and upper limit (i.e., ±1.5×(Q3−Q1)).

Given the probabilistic forecasts, one can assess the reserve requirements computed by the IR service. As explained in the STAR Methods sections, a convolution process on the probabilistic forecasts, plus additional considerations on other system variables (conventional generation, imports, exports, and pumping), allow us to compute the System Margin. From the System Margin, risk indices such as the Loss of Load Probability (LOLP) and Probability of Curtailing Renewable Energy (PCRE) are calculated and represented through risk-reserve curves. Given a certain risk threshold, it is possible to calculate the upward and downward reserve requirements. This process is repeated for each country and hour of the day. An example of this exercise is available in Figure 2 for Portugal. The RES and load quantiles are plotted, and the computed system margin is approximately centered around zero, as expected in a balanced system, but with a larger tail to the left side. This results in an upward reserve requirement ($R_{up}$), calculated from the LOLP, being superior to the upward deterministic rule of reserve ($DRR_{up}$). In this case, a recommendation for consumers to decrease their consumption is expected for this period to mitigate the possible scenario of loss of load. In a more aggregated manner for the whole period, Figure 3 reveals, by hour of the day, the reserve requirements and the current deterministic rule for operating reserve only for periods where electricity consumption increase or decrease recommendations were issued for Portugal.

**Figure 4:**
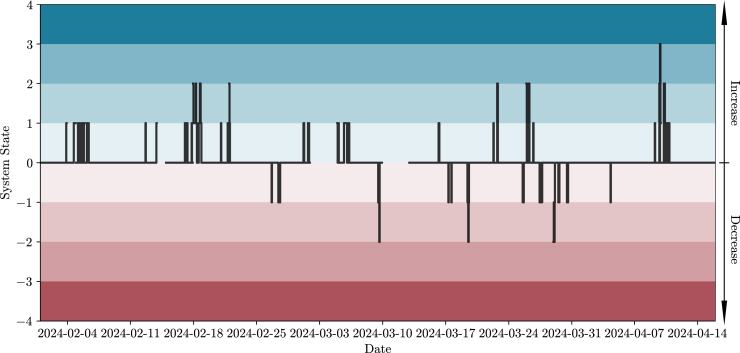
System State for Portugal A time series of the System State (a black line ranging between −4 and 4) for Portugal for the whole period of analysis. When the System State is above 0, consumers are expected to increase their electricity consumption (different magnitudes are related to stronger shades of the color blue). When the System State is below 0, consumers are expected to decrease their electricity consumption (different magnitudes are related to stronger shades of the color red). If the System State is 0, no action is expected from consumers.

**Figure 5:**
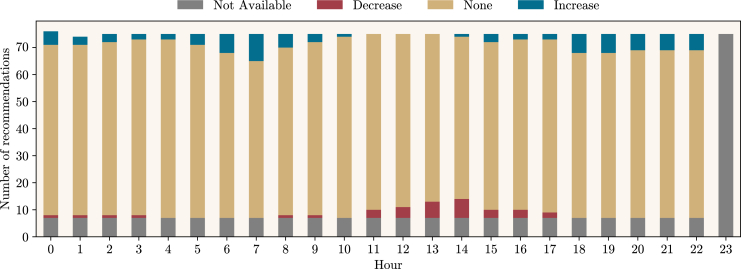
Histogram of recommended actions for Portugal The number of actionable recommendations provided to consumers during the period of analysis is depicted through a stacked histogram according to its category and related to the hour of the day for Portugal. Recommendations to increase and decrease consumption are available, respectively, in blue and red. When the country is *healthy*, no recommendation is provided (plotted as None, in yellow). When any data is missing, no recommendations are calculated (Not Available, in gray). Recommendations to decrease consumption are more frequent between hours 11 and 17, while for hour 23, there were never recommendations available because of missing data (specifically due to the timezone difference between Portugal and Spain).

Next, an analysis of the recommended actions for Portugal during the mentioned period is conducted. Figure 4 depicts a time series of the System State (between −4 and 4, a scale related to the magnitude of the recommendation, further defined in the STAR Methods), while Figure 5 performs an analysis of recommended actions by hour of day. These figures indicate that there are few recommendations for Portugal; they occur in both directions (increase or decrease), and it is uncommon for them to lead to a System State of 3 in any direction. Furthermore, the figures indicate that recommendations to reduce consumption are primarily concentrated between hours 11 and 17. An overview of the provided recommendations for the whole period shows that less than 7% of the total number of hours included a recommendation to increase or decrease consumption. Note that recommendations can be the result of risk in a specific country or in one of its neighboring countries. In this case, the results indicate that recommendations for Portugal are solely due to risks identified within the country’s own risk indices rather than its interconnection with Spain.

**Figure 6:**
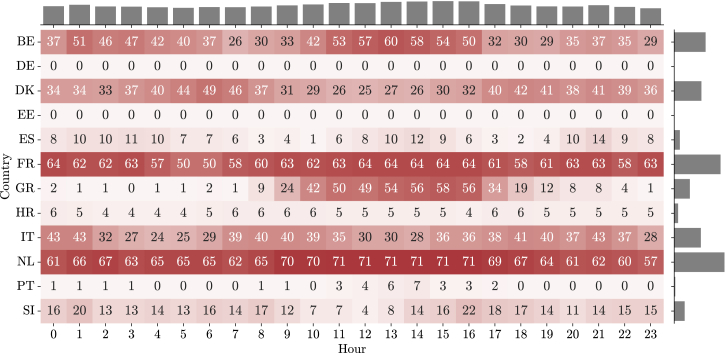
Decrease actions by country and hour of day An overview of the number of *decrease* recommendations provided to consumers of the participating countries by hour of day. A heatmap with different shades of red relates to the total number of alerts generated. The plot also depicts the marginal histograms with the total per hour of day or total per country.

**Figure 7:**
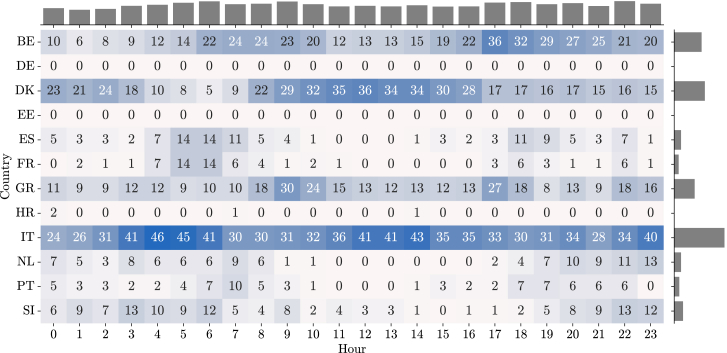
Increase actions by country and hour of day An overview of the number of *increase* recommendations provided to consumers of the participating countries by the hour-of-day. A heatmap with different shades of blue relates to the total number of alerts generated. The plot also depicts the marginal histograms (in gray) with the total per hour of day or total per country.

A high-level analysis can be performed at the European level. An interesting visual output of the service could be a map of Europe that overlaps with the recommendations but only depicts a specific hour. To assess the recommendations for all the countries for a longer period of time, a heatmap combined with marginal histograms provides an idea of the hours of the day and countries that have the most recommended actions. Figures 6 and 7 showcase the number of recommendations to, respectively, decrease and increase consumption for the countries that were part of the InterConnect project. During this period, a total of 9574 *increase* or *decrease* recommendations were issued for all countries (72% to decrease, and 28% to increase). The marginal histograms make clear that recommendations to decrease consumption are, overall, concentrated between the late morning and early afternoon period, while the number of recommendations to increase consumption is higher during the early morning and mid/late afternoon period.

**Figure 8:**
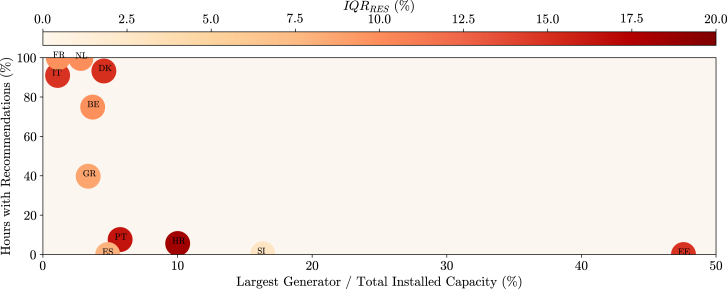
Relationship between largest generator and number of recommendations The share that the largest generator of a country represents in terms of its total generation capacity is related to the number of hours in which it is *at risk* and, thus, the total number of *increase* or *decrease* recommendations. This happens because the higher the share, the larger the deterministic rule for the reserve, and therefore, it is less likely that the reserve requirements surpass the deterministic rule. This relationship is verified in the Figure with the participating countries. As a complement, the Interquantile Range (IQR) metric for the probabilistic RES forecasts is plotted for each country by a circle that ranges from yellow (lower error) to red (higher error).

As further explained in the STAR Methods, a country’s System State depends on a deterministic rule for the reserves (DRR). This rule considers a reference for the manual frequency restoration reserve (mFRR) and replacement reserve (RR), the loss of the largest generation unit connected to the electrical network, and two error terms related to the mean absolute percentage error of the generation and load forecasts. Therefore, it is possible that countries that have their largest generator representing a higher share of their total installed generation capacity can have a lower number of recommendations. This stems from the fact that, for countries with roughly the same total generation capacity, having a larger “largest generator” ($G_{c}^{largest}$) leads to a higher deterministic rule and, therefore, a lower probability of having risk indices that surpass the pre-defined risk threshold. This relationship can be verified in Figure 8, where there is a clear relationship between the share of the largest generator and the number of hours with increase or decrease recommendations. To compute this Figure, only hours without missing data were considered, and recommendations unrelated to interconnections were included.

Moreover, countries, where the probabilistic forecasting of RES or load is more uncertain (e.g., with a higher IQR metric), are expected to reveal longer tails on the probability distribution of the system margin, ultimately leading to higher reserve requirements. This uncertainty can contribute to a larger number of hours with recommendations, although Figure 8 suggests that the share of the largest generator is the dominant factor. Using country-specific rules to calculate the DRR, tailored to the details of how each transmission system operator (TSO) determines the reserve requirements, could help overcome this issue. However, this information is difficult to collect as it is often not available in English, not in a structured format, or not publicly accessible. In the future, large language models could assist this task by organizing unstructured data and identifying and summarizing relevant publicly available information, making it more accessible and useful.

### Insights from the mobile energy applications demonstration

This subsection presents the results of the real-world demonstration of the IR service integrated with third-party mobile energy applications (illustrating the practical application of semantic interoperability principles) conducted within the framework of the large-scale pilots from the Inter Connect project. The focus is the pilot in Portugal, which can be considered the *lighthouse* pilot where the IR service was first tested and benchmarked, and the ones in Greece and Croatia as *follower* pilots, which adopted and adapted the services demonstrated by the *lighthouse* project.

The pilots relied on the IR service to provide country-specific recommendations as a signal to consumers. This allowed different settings and countries to be included in the demonstrations, which can lead to different interpretations of recommendations provided by the IR service. Through a backend named Energy App, a single instance of the IR service provided the recommendations for all the countries. The recommendations were provided in an interoperable manner (i.e., through the SIF), which can be leveraged by third parties (i.e., the promoters of the pilot projects) as shown in the STAR Methods section.

**Figure 9:**
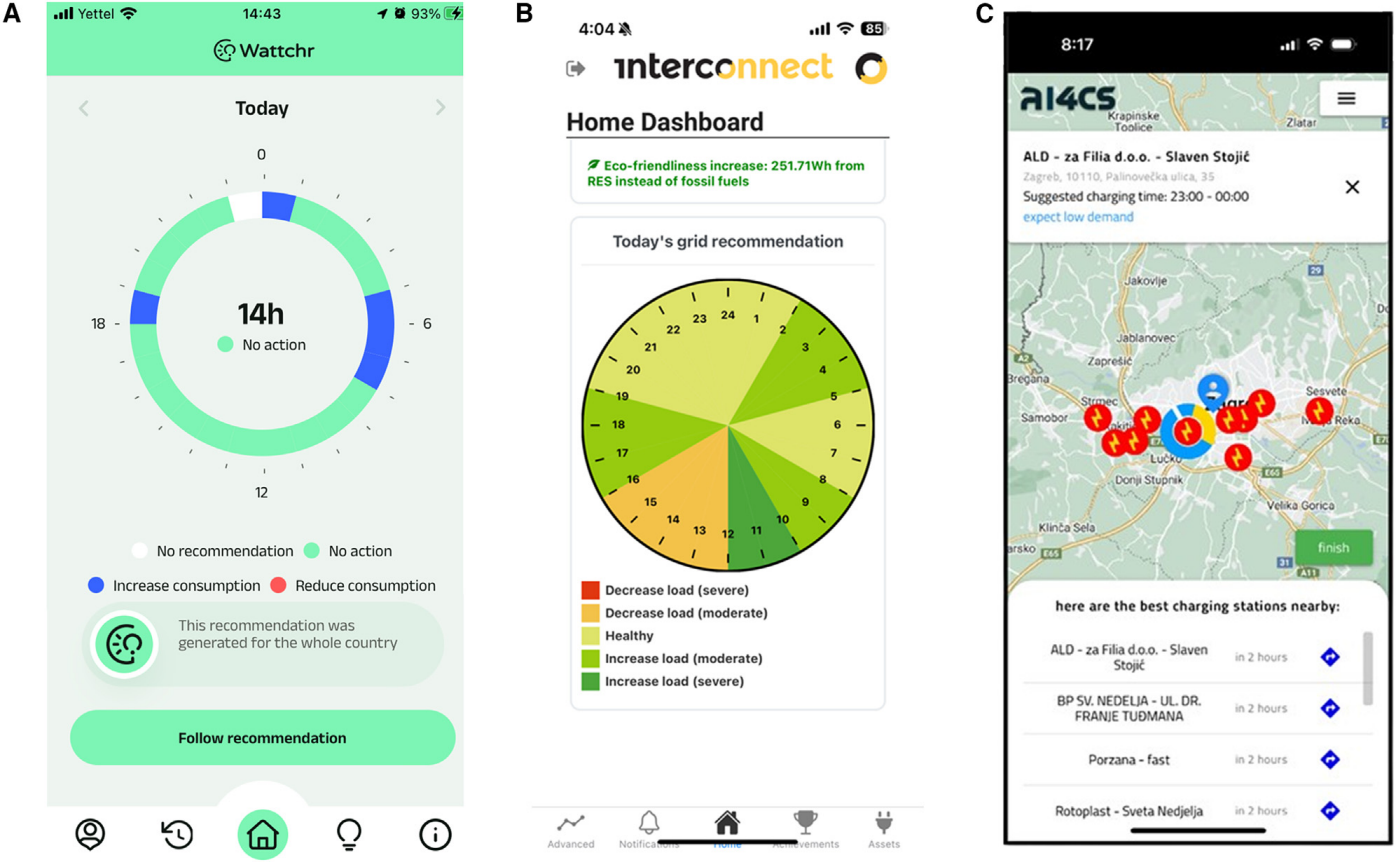
Mobile applications from pilot projects (A–C) depict mobile applications developed or improved within the context of the InterConnect project to support pilots and communicate recommendations to consumers. The preferred visualization of the recommendations was a clock-like structure with periods to increase or decrease consumption. (A) is the *Wattchr* app for the Portuguese pilot, which handles recommendations from the IR service and the DSOi and communicates with residential consumers. (B) is the app for the Greek pilot, which handles recommendations from the IR service and the DSOi and communicates with residential consumers. (C) is the app for the Croatian pilot, which handles recommendations from the IR service and communicates with EV drivers from the local charging network operator.

Three distinct examples of demonstrators were portrayed in this section, summarized in Table 2. The table also presents the start and end dates of the pilots, the tools used to generate recommendations, the IR and the DSO Interface (DSOi), and the number of participants who registered to receive recommendations. An example of the mobile application screens is presented in Figure 9.

**Table 2:** Summary of pilot project information

	Country	Setting	IR	DSOi	Start	End	Participants
Portuguese pilot	PT	Residential	✓	✓	2023-08-08	2024-05-31	68
Greek pilot	GR	Residential	✓	✓	2024-01-05	2024-05-31	150
Croatian pilot	HR	E-mobility	✓	X	2024-01-16	2024-02-20	112

#### Portuguese pilot

The Portuguese pilot relied on four key components: the IR service, the DSOi, the Energy App backend, and a mobile app for the end user named *Wattchr*. During the pilot project, the IR service generated alerts that advised consumers on the optimal periods to increase, decrease, or maintain their electricity consumption.

Since the IR signals might not consider the limitations of local distribution networks at medium or low voltage levels, the DSO must also avoid technical problems from DR actions, such as overloads from simultaneous load increases in the same network area for balancing purposes. To mitigate these issues, the DSO can refine the IR signals to account for the capacity and operational constraints of the local distribution network. Therefore, the IR alerts were specialized with the DSOi, based on the zip code of consumers included in the pilot project. Further details on the DSOi and the specialization process (the available methods and their priorities) are available in the STAR Methods section.

The *Wattchr* mobile application provided the means for consumers to access the recommendations and further characterize the energy resources available in their households. Moreover, the *Wattchr* app educates consumers by sending daily good practices that contribute to more sustainable energy usage. *Wattchr* was distributed to the participants of the Portuguese demonstration, most of them located in the north of the country, namely in the city of Braga. The majority of the participants live in apartments or single-family homes with an average household composition of two adults and one child. Participants were engaged through the overall piloting activities within the Portuguese pilot of the InterConnect project as clients of the electricity supply company that is affiliated with the Portuguese DSO or as members or employees of companies that are part of this project. This group first included 17 residential customers (*beta testers*). In the final weeks of the testing phase, *Wattchr* was made available to approximately 250 residential pilot participants as an optional app that they could use to contribute to the CERF results. By March 31^*st*^ of 2024, *Wattchr* held 68 registered users. The mobile app displays the recommendations with a clock-like structure, highlighting the periods when consumers should increase or decrease their energy consumption, as depicted in Figure 9.

To support the interpretation of the specialization process (further explained in the STAR Methods), an example of the *Automatic* specialization method is presented. On the $6^{th}$ of December 2023, the IR signal for Portugal was retrieved by the DSOi for the next day, with a risk evaluation of *increase* for 4 a.m. The DSOi changed the risk evaluation for a specific zip code from *increase* to *healthy*. However, the original risk evaluation was kept for the majority of the Portuguese territory. This specialization occurred due to a forecasted high power transformer capacity usage of 83.2% (i.e., the ratio between the forecasted electrical load and the transformer’s rated capacity) for the primary substation serving the electrical loads within this zip code. This usage would exceed the predefined 80% threshold established by the *Automatic* method. Therefore, consumers in this zip code were advised not to increase their electricity consumption during this period, to respect the local technical network constraints.

An example of the *Semi-automatic* specialization method is also presented, which incorporates additional features of the DSOi, namely the Flexibility Management Module. On the $6^{th}$ of February 2024, the IR signal was retrieved by the DSOi for the next day, with a risk evaluation of *healthy* for 7 p.m., specifically for Portugal. For a specific zip code, the DSOi changed the risk evaluation from *healthy* to *decrease*. This change occurred due to a need for flexibility that was identified by the planning tool, which demanded a 100 kW load reduction in an area that included the mentioned zip code.

Given these examples, Table 3 summarizes the number of recommendations provided by the IR service for different months and the impact of deploying the DSOi to specialize them. For example, in January of 2024, the IR service provided a total of 621 recommendations (*healthy*, *increase* or *decrease*) at the country level for Portugal. The DSOi retrieved those 621 recommendations and provided 226778 to consumers after disaggregating them by all the zip codes involved in the demonstration. Each of those was specialized through one of the available methods (*Automatic*, *Semi-automatic*, or *Manual*), which can keep or change the recommendation. For the mentioned month, 96.66% (219213) went through the *automatic* and 3.32% (7533) through the *semi-automatic* specialization process, with 0.01% (32) being *manually* specialized. Still, only 3.17% (7193) of the total recommendations were changed from their original category.

**Table 3:** Recommendations and feedback provided for the Portuguese pilot

	11/23	12/23	01/24	02/24	03/24	Total
**IR Recommendations**						

Total (hourly)	619	659	621	345	69	2313

**DSOi Recommendations**						

Total (hourly, all zip-codes)	61612	239971	226778	124017	24636	677014
Specialization: *Automatic* (%)	99.25	96.94	96.66	98.20	98.87	97.36
Specialization: *Semi-automatic* (%)	0.74	3.06	3.32	1.80	1.13	2.64
Specialization: *Manual* (%)	0.01	0.00	0.01	0.00	0.00	0.01
Recommendations changed (%)	0.30	3.49	3.17	1.80	1.13	2.69

**Mobile App Feedback**						

★☆☆☆	2	14	17	4	3	40
★★☆☆☆	0	1	1	0	2	4
★★★☆☆	4	3	11	0	5	23
★★★★☆	2	9	17	2	9	39
★★★★★	45	38	63	49	64	259

**Figure 10:**
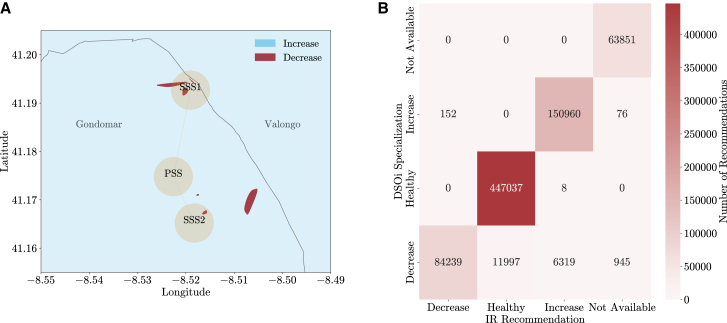
DSOi specialization of recommendations (A) An example for December $7^{th}$ of 2023, at 4 a.m., where specific zip codes in Portugal were asked to decrease consumption (patches in red), while the country-level recommendation was to increase (blue). This occurrence was due to the *Automatic* specialization process of recommendations. In this case, one of the nearby secondary substations (the primary – PSS – and secondary – SSS1 and SSS2 – substations associated with the zip codes were plotted for support in orange) was overloaded (i.e., was over the pre-defined 80% threshold of capacity usage). Therefore, to comply with local network constraints, the recommendation was changed. (B) A heatmap enables a better understanding of what are the main specialization paths (e.g., the total number of recommendations being changed from *healthy* to *decrease*) under the different specialization processes available by the DSOi.

Figure 10 presents an example of specialization for specific zip codes, including the geospatial data of relevant electrical network assets. For December $7^{th}$ of 2023, at 4 a.m., the IR service recommended consumers to increase their consumption. However, after the DSOi specialization, a few zip codes in one of the northern municipalities changed from *increase* to *decrease*. More specifically, the *Automatic* specialization method was used, given that a nearby Primary Substation (*PSS* in the Figure) is connected to two Secondary Substations (*SSS1* and *SSS2* in the Figure), and one of those is usually overload (80–99% used capacity, the threshold being 80%). Overall, the change from one action to another is also captured in Figure 10, where it is clear that only a few actions changed from the original ones provided by the IR service.

During the pilot period, the *Wattchr* application gathered daily user feedback on the likelihood of adopting the provided recommendations by asking them to adopt a one to five-star rating scheme, respectively from *Very Unlikely* to *Very Likely*. Table 3 depicts the results by month, revealing that, in total, most users opted to vote the highest rating (4 and 5 stars), a total of 298 out of 365 (81.6%), showing commitment to adopt actions that contribute the most to each hourly recommendation issued.

The results were also assessed in terms of the impact that the *increase* or *decrease* actions had, as some of the participants in the pilot had metering data available from the DSO. Assessing this impact is challenging and is also discussed in the context of Flex Alert,^31^ where authors state that there is no ground truth on what the demand would have been in the absence of a specific DR signal and develop reference cases for comparison. A similar approach was taken in this work where, for each day with recommendations, a set of comparable days were retrieved, obeying the following criteria: a) the comparable days must be within a 15-day range of the day being analyzed (target day), b) the comparable days must be of the same type (weekday/weekend) as the target day, c) the comparable days must not be targeted with any *increase* or *decrease* recommendations, and d) only the three comparable days with the closest daily average temperature to the target day were kept.

**Figure 11:**
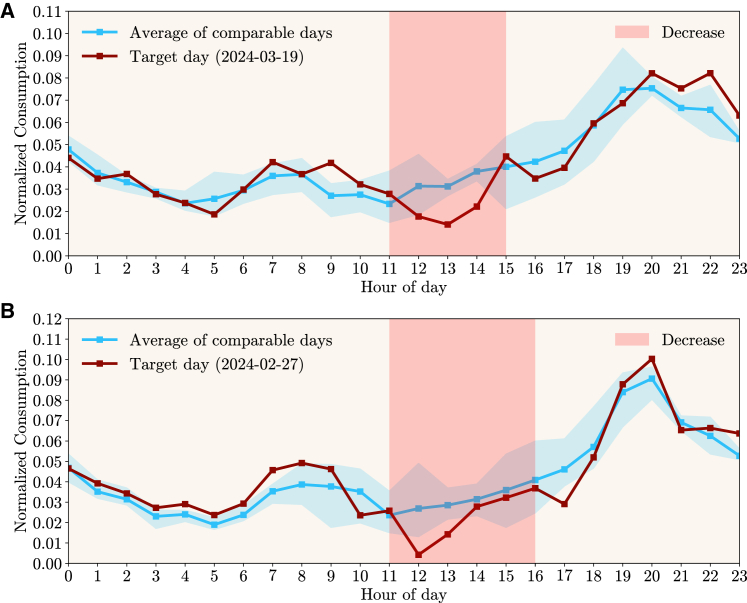
Portuguese pilot: Comparison between days with recommendations to decrease consumption and historically comparable days (A and B) represent the normalized consumption profile (red line) of a target day that had multiple consecutive hours with recommendations to decrease consumption (shaded region in light red). It also shows the average of the three historical days (blue line) that are comparable according to a set of defined criteria and the full range of those three days (shaded region in light blue). (A) Represents the Target day of $19^{th}$ of March of 2024, while (B) shows the Target day $27^{th}$ of February of 2024. The *Flex Period Response* metric for these days is −4.2%.

For each day, the hourly consumption of twenty-five consumers was added and then normalized. To illustrate the approach more clearly, only days that had a single period of multiple hours with a recommendation in one direction (*increase* or *decrease*) are shown. The *Flex Period Response* metric (Equations 2, 3, and 4 of Peplinski and Sanders^31^) was computed, which evaluates the change in the normalized electricity demand profile during the period where a signal was communicated to consumers in contrast with the average normalized profile of the comparable days for the same period. Figure 11 shows data for the $19^{th}$ of March 2024, where consumers were recommended to decrease their consumption between hours 11h00 and 15h00. The normalized consumption of this day differs slightly from the average of the three comparable days, with a larger difference showing in the period with a recommendation to decrease. Overall, the percentage of demand occurring within the recommendation period went from 12.4% on the comparable days to 8.2% on the day with the recommendation, resulting in a −4.2% *Flex Period Response*. The same Figure also presents another example, for the $27^{th}$ of February 2024, where the normalized consumption reveals a more pronounced decrease in hour 12h00. On that day, the percentage of demand within the recommendation period went from 14.6% to 10.4%, resulting in a *Flex Period Response* of −4.2%.

**Figure 12:**
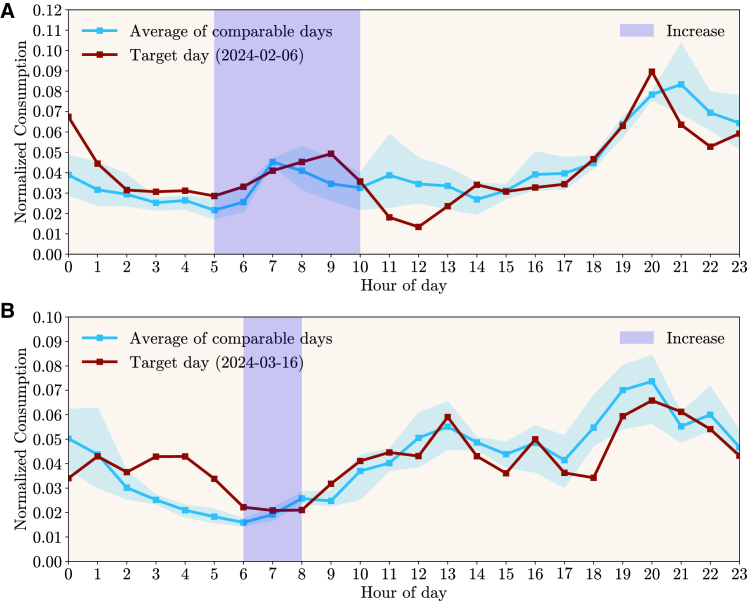
Portuguese pilot: Comparison between days with recommendations to increase consumption and historically comparable days (A and B) represent the normalized consumption profile (red line) of a target day that had multiple consecutive hours with recommendations to increase consumption (shaded region in blue). It also shows the average of the three historical days (blue line) that are comparable according to a set of defined criteria and the full range of those three days (shaded region in light blue). (A) Represents the Target day of $6^{th}$ of February of 2024, while (B) shows the Target day $16^{th}$ of March of 2024. The *Flex Period Response* metric for these days is, respectively, +2.9% and +0.8%.

For the case of recommendations to increase consumption, Figure 12 reveals two days ($6^{th}$ of February and $16^{th}$ of March of 2024) where a single period of multiple hours with a recommendation was provided. The *Flex Period Response* metric was computed using the same method, resulting in, respectively, +2.9% and +0.8%. For the days analyzed, consumers were less prone to comply with *increase* than *decrease* recommendations. However, this could be due to different factors that were not accounted for, and these *increase* recommendations were set in the early morning, which impacts the participation of consumers who adjust their consumption manually.

#### Greek pilot

The Greek pilot enrolled approximately 150 residential consumers from three major cities: Athens, Thessaloniki, and Volos. The majority were homeowners (70.97%), while the rest (29.03%) were tenants. They came from various professional backgrounds, including professionals, office workers, and retirees, with a large portion (43.33%) identifying as professionals. Regarding education, 50% held university degrees, 30% had completed postgraduate studies, and 6.67% had earned doctoral degrees. Most participants were between the ages of 36 and 55, and their household compositions ranged from singles to families. Participant involvement in the project was primarily due to their direct connection as employees of companies participating in the pilot program, with additional participants joining through referrals from relatives and friends. A large majority (83.33%) lived in apartments, while the remaining 16.67% resided in other types of housing, such as detached homes or townhouses, reflecting a diverse range of living conditions. Their participation in the pilot involved an onboarding process where smart meters were installed in their homes for continuous energy monitoring. Additionally, they were equipped with IoT gateways and sensors for monitoring indoor/outdoor temperature and humidity, human presence, and door status. Half of the residences were outfitted with smart appliances that could receive commands to adjust their operating schedules based on DR schemes. After installing new devices, some existing devices were retrofitted to smart versions, such as electric water heaters that were upgraded for remote operation.

Several services were developed to validate use cases related to energy and smart home services. The main goal was to leverage new technologies for real-time monitoring and action based on an extended dataset that included energy readings from the smart meter and additional sensory information about each residence’s indoor and outdoor environments. The SIF ensured data and service interoperability to facilitate the exchange of heterogeneous data and APIs among various stakeholders, from ICT small and medium-sized enterprises (SMEs) and telecom providers to energy utilities. The services implemented offered capabilities such as DR, personalized recommendations, and a user interface built on backend services such as live data from energy and sensors, forecasting, and third-party sources such as TSO data.

For the purpose of user engagement and bi-directional exchange of information between the pilot services and participants, a mobile app was developed to act as a user interface for end users through appropriate engagement mechanisms, such as real-time data visualization, gamification, push notifications, device actuation, and feedback provision.^46^ The mobile app is fully interoperable by integrating the SIF to facilitate a unified user interface for consumers from different IoT service providers and energy retailers, interconnecting their backend systems in a semantically interoperable manner. For example, end-users can log in to the mobile app, which can handle users coming from multiple SAREFized IoT platforms of the Greek pilot.

Specifically, after logging into the mobile app, the user is greeted by the home dashboard, which is the initial screen providing insights into the household’s energy usage. The dashboard updates daily with recommendations, displays comparative energy consumption data for today and yesterday, and a summary of the total daily energy use over the past week. The inventory overview screen lists all household devices, some of which can be managed remotely through the app and allows users to set their flexibility preferences for DR actions. The gamification screen showcases the user’s progress in various achievements and provides feedback opportunities for notifications received. For in-depth analysis, the advanced dashboard offers historical data analytics, where the user can examine specific measurements and time slots for all connected devices to review past data trends. Lastly, the push notifications and feedback screen displays all notifications received, allowing the user to respond with acceptance or rejection.

Building on top of this interface, the app integrates with the IR service, which provides hourly recommendations on a daily basis. Within the mobile app, a dashboard was used to present these recommendations, visualized on a 24-h clock format, as depicted in Figure 9. Each hour of the day was color-coded to indicate recommended actions to *reduce* or *increase* energy consumption, with a moderate or severe level, a scale based on the *risk_level* output of the IR. These recommendations were presented on the app’s initial screen, alongside consumption information and a secondary, personalized recommendation based on the forecasted peak consumption time of the household. Recommendations were tailored with data from the ENTSO-E Transparency Platform, enhanced by the DSOi, and linked to specific data about transformers from the Greek DSO associated with the zip codes of participants. Although the DSOi was implemented, no specialized recommendations were issued to consumers during the period of the pilot project.

Feedback from six participants through surveys and interviews indicated a positive interest in understanding how their energy consumption impacts the electrical network. Five of the six participants interviewed reported interacting with the mobile application at least once a day but suggested more visual aids for a better understanding of the recommendations. Four out of six participants confirmed that their energy usage was being adjusted to the recommendations but expressed only a moderate understanding of how their actions could impact the overall health of the electrical network. Finally, according to the daily recommendations, the absence of economic incentives was reported as a barrier to changing their energy consumption habits. These conclusions open new research opportunities, for example, to link the recommendations with a dynamic tariff scheme and test the potential to motivate consumer behavior change through pricing and how it compares to voluntary DR.

**Figure 13:**
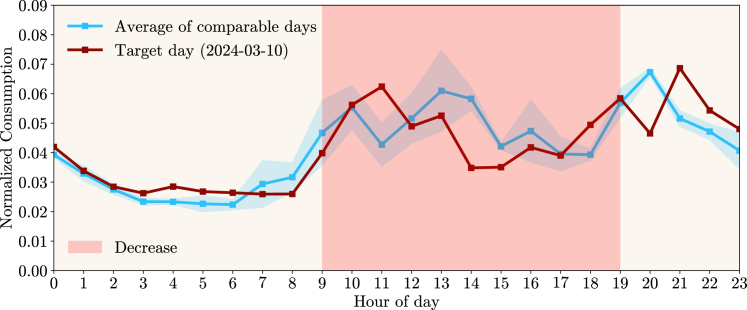
Greek pilot: Comparison between days with recommendations and historically comparable days This Figure represents the normalized consumption profile (red line) of a target day ($10^{th}$ of March of 2024) that had multiple consecutive hours with recommendations to decrease consumption (shaded region in light red). It also shows the average of two historical days (blue line) that are comparable according to a set of defined criteria and the full range of those two days (shaded region in light blue). The *Flex Period Response* metric for this day is −2.4%.

Metering data from thirty-three households that participated in the Greek pilot were acquired to analyze the impact of the *increase* or *decrease* actions on household electricity consumption. An exercise identical to the one made for the Portuguese pilot was conducted by comparing target days with comparable days that followed specific criteria. Figure 13 depicts the results of the exercise for one target day. Several reasons that are specific to how the Greek pilot was conceptualized can justify why it is not clear that conclusions similar to the ones for the Portuguese pilot could be drawn.

First, the Greek pilot provided not one but two simultaneous signals to the consumers (one from IR outputs and one related to shifting peak consumption to low-carbon periods), which complicates the process of assessing the outcomes since it is not clear how to separate the effect of each signal. This phenomenon can also be related to a concept known in the literature as response fatigue,^47^ where, over time, the initial enthusiasm or responsiveness to DR programs decreases, leading to a gradual return to “default” consumption behaviors. This underscores the importance of presenting information to consumers clearly and straightforwardly. Overloading consumers with multiple signals or complex data can be counterproductive, leading to confusion and reduced engagement.

Second, the IR performance for Greece (Figures 6 and 7) shows that the country had many hours with recommendations, both to increase and decrease consumption. This means that there are only a few days where no recommendations were set (which would be possible candidates for comparable days), but also a few days where only one of the directions (*increase* or *decrease*) was available for consecutive hours (which would be possible candidates for target days).

For the target day presented in Figure 13, the *Flex Period Response* was −2.4%. Still, only two comparable days were found (even when loosening the 15-day range), while three were requested by the methodology. Overall, it was concluded that the applied methodology is not ideal for countries with many recommendations and different increase/decrease directions on the same day.

#### Croatian pilot

Multiple third-party services (i.e., using the cascading funding from the InterConnect project for start-ups and SMEs) used the IR service outputs as a non-economic signal for consumers to adapt their energy consumption behavior. A clear example is the AI4CS (*AI for Charging Stations*) digital service, developed by Local AI (a technology startup) for electrical mobility.^48^ The company conducted a pilot in Croatia in collaboration with Hrvatski Telekom, the largest EV Charging Stations Network Operator in the country.

The AI4CS platform initially focused on forecasting the consumption patterns of EV charging station networks to plan for expansion and assist end-users in locating available charging stations through a mobile and web application. These forecasts were generated using state-of-the-art algorithms, including transformers and long short-term memory (LSTM) architectures, which predict the day-ahead hourly availability of each charging station. The algorithms were trained on charging session data provided by the network operator.

The pilot consisted of demonstrating a mobile application that guides EV owners to public charging stations in specific locations and periods to improve user experience and convenience for EV charging (avoiding periods of high probability of occupied EV chargers) while accounting for country-level network resilience concerns. Therefore, based on forecasted availability and the network-level signals coming from the IR service, the AI4CS application promoted users to change their usual EV charging habits, suggesting charging in hourly periods that are scored as *healthy* or *increase* by the IR service, while simultaneously discouraging them from charging at *decrease* periods. The application requests daily day-ahead recommendations from the IR service via the Energy App’s backend, using the SIF, and suggests a charging station resulting from a matching/scoring algorithm.

EV drivers can use the mobile application to get a suggestion of where and when to charge with the following steps: i) the application asks the user to give a location or alternatively to enable the use of their current location; ii) the user is asked for a maximum driving distance from the selected location, and the application generates the equidistant polygons for all the kilometric options allowed; iii) the user selects the best hour for charging according to his preference; iv) the application suggests an optimal charging station, located within the specified driving distance, to charge in a period within 2 h of the preferred one; v) the application displays the name, distance, and driving directions to the suggested charging station; vi) finally, the user is asked to provide feedback on user experience.

To make EV drivers aware of the recommendations, the application overlaps the IR outputs, graphically depicted as a doughnut chart of the hourly recommendations, on top of geospatial data (i.e., the location of the multiple charging stations available on a map). An example of the interface displayed to EV drivers is depicted in Figure 9.

The pilot project included 112 participants (EV drivers) who regularly used the application between January $16^{th}$ and February $20^{th}$ of 2024. A random subset of the customer base of Hrvatski Telekom was approached to become participants of the pilot, and it was ensured that the gender and area of residence of the participants were represented in a proportional manner. Since the pilot consists of EV owners, it typically includes people from middle to higher incomes in Croatia due to the current penetration profile of EVs. During this period, logs related to its usage were collected from the AI4CS application’s backend, along with immediate feedback from the users regarding each suggestion provided.

**Figure 14:**
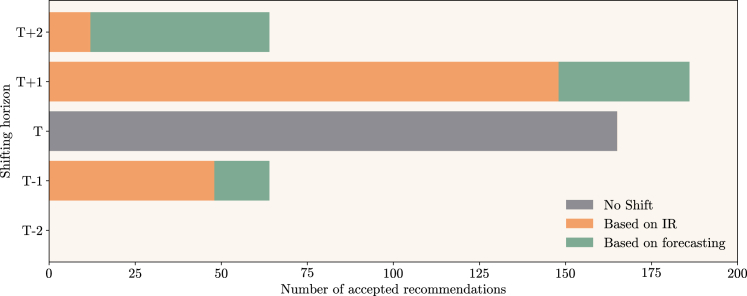
EV charging session shifting based on AI4CS suggestions The AI4CS platform delivered suggestions for EV drivers to charge in specific periods of the day, from T−2 to T+2 of their preferred charging time *T*. Those suggestions can be based on the charging stations usage forecasting (depicted in green) or on the IR service country-level recommendations for Croatia (depicted in orange). From the 480 accepted suggestions, 208 were delivered based on the outputs of the IR service, and EV drivers were mostly willing to postpone their charging session by 1-h relative to their preference.

It was found that, for the mentioned period, 480 suggestions requested from EV drivers were accepted, from which 208 (43%) were determined by the IR outputs, suggesting a shifting in the charging process within a 2-h period around the users’ preferences and constrained by the maximum driving distance. Regarding the shifting, Figure 14 shows that EV drivers were more inclined to postpone their charging session to 1 h later (T+1) or keep it at the preferred hour (*T*). For time *T*, the suggestions matched with the initial requests of the users’ preferred charging hour since the expected usage of the nearby stations was low, and the IR service did not recommend a *decrease* action in the same period. The number of accepted suggestions to anticipate a charging session by 1 h (T−1) or to postpone it by 2 h (T+2) was similar. Furthermore, there were no accepted suggestions to anticipate charging by 2 h (T−2).

Regarding the application itself, user satisfaction was measured resorting to the Mean Opinion Score (further characterized by Streijl et al.^49^), reaching 3.8 on a scale of 1 (Bad) to 5 (Excellent) and the Net Promoter Score (further characterized by F. Reichheld^50^) of 7.5 on a scale of 1 (Not recommend at all) to 10 (Strongly recommend). The code for charging station availability forecasting and for the mobile application and the application logs from the pilot period can be found in the data and code availability section.

## Discussion

In this work, the IR service provided recommendations (actions to increase or decrease consumption) to pilot programs in twelve European countries, and results for three pilots were discussed.

The alerts given to consumers differ from similar solutions such as Flex Alert, as *increase* actions are also delivered when excess RES-based generation is expected to be curtailed. Peplinski and Sanders^31^ report some specific cases where voluntary actions were effective in an emergency context. The IR service computes recommendations that not only target emergency situations directly but also attempt to mitigate the predicted imbalance of each country on a daily basis and local network constraints when coupled with the DSOi service.

It was concluded that, during the period of analysis of the IR service, Portugal accumulated recommendations both to *decrease* and *increase* consumption, reaching a maximum System State of three (out of four). Regarding *decrease* actions, they were mostly concentrated between hours 11 and 17 and occurred during 2% of the total hours.

From the specialization process done by the DSOi, exemplified for Portugal, it was shown that only a small percentage of the total number of recommendations were changed in terms of category during the pilot period. We can, therefore, conclude that only a small set of recommendations provided by the IR service were, in some way, against the expected logic of the DSO and its challenges. It is also clear that the *Automatic* method, although it is the one with the lowest priority, is the one that most of the recommendations go through. Specializing in IR signals was a means to couple the country- and local-level challenges, prioritizing issues at the distribution network level when needed and managing recommended actions to deal with possibly conflicting objectives.

Metering data from twenty-five consumers included in the Portuguese pilot were retrieved from the pilot project period. By comparing their aggregated consumption on specific days that contained recommendations with comparable days that obeyed certain criteria, it was found that a *Flex Period Response* of around −4% was achieved on some days. As there is no ground truth to compare, this conclusion needs further validation and should rely on data from more consumers. Considering socio-demographic information and resorting to causal models could improve the insights into the reaction of consumers to voluntary DR programs, for example, by computing counterfactuals.

Regarding the Greek pilot, five of the six interviewed participants reported a daily usage of the mobile application, with four confirming an adapted energy usage as a response to the provided signals. Metering data from thirty-three households were analyzed, but no conclusions were drawn due to multiple signals being provided to consumers simultaneously.

From the Croatian pilot, 27% of the 480 accepted suggestions to charge at specific periods were defined by the IR service output, and it was concluded that EV drivers were more willing to postpone their charging session by 1 h than to anticipate it. It was also concluded that the number of accepted suggestions to anticipate a charging session by 1 h or to postpone it by 2 h was similar.

Both the Portuguese and Greek pilots were directed to the residential sector. In contrast, the case presented in the Croatian pilot was related to electric mobility, which showed diversity in the context where the IR can be used. The characterization of the different pilot projects showed how the IR service can be used with different levels of detail. For example, third-party services can include or not include the DSOi specialization and include or not include the magnitude of the recommendations.

Overall, this work shows that it is feasible to implement a voluntary DR program at the European level by promoting actionable recommendations to consumers, which include information on the local network and the interconnections between the countries. Furthermore, it demonstrated the operation of the service considering only open data sources.

### Limitations of the study

Many limitations can be noted in the present study. Data availability poses a challenge that is difficult to overcome (e.g., due to missing and incorrect data from data providers) as only one data source is being used. Consequently, the hour at which the tool is executed is dependent on data providers complying with ENTSO-E deadlines or providing the data in a timely manner to enable day-ahead notice.

On another note, the methodology does not consider country-specific rules to set the requirements for operational reserves; it is only a general one. The methodology also does not consider the simultaneous effect of all mitigation actions from neighboring countries nor the full impact on their electrical system, which could be overcome with more complex representations of each country’s electrical system. Moreover, the recommended actions do not quantify how much the load should increase or decrease. Therefore, further improvements in recommendations could benefit from a continuous feedback loop from consumers, estimating their change in energy consumption with real data. A future quantitative recommendation system (i.e., including how much consumers should increase or decrease their energy consumption) can target specific consumers according to their sociodemographic information or other drivers^31^ (e.g., outdoor temperature, flexible schedules, health, age, among others). This system, when coupled with a learning process on consumers’ response to the recommendations, would avoid increasing or decreasing consumption by too large of a margin, improving the efficiency of the DR program. Ultimately, understanding the willingness of individual consumers to alter their energy consumption behavior in specific periods of the day allows system operators and utilities to overcome not only day-ahead predicted system imbalances by adjusting consumption at the national level but also to overcome or prevent issues at the local distribution level, by adjusting consumption at specific regions or zip-codes, enhancing the overall resilience of the system.

Many complications arise from the large-scale implementation of a service such as the IR. Maintaining an adequate infrastructure and access to consumer metering data becomes challenging as the number of consumers enrolled in the DR program increases. Data privacy concerns from recent regulations such as the General Data Protection Regulation (GDPR) enforce limitations when accessing and processing sociodemographic information of individual consumers. Providing consumers with a feedback mechanism is paramount to assess the effectiveness of the DR program, but asking for regular and detailed feedback can increase the cognitive load of consumers. The availability of sociodemographic and household information was limited in this study, and only a simple daily feedback mechanism was implemented. Therefore, it was not possible to directly correlate the individual feedback of consumers and the amount or type of their smart appliances with their metering data. Consequently, only a high-level analysis was performed on an aggregated level.

Future work includes improving how recommendations are set to neighboring countries with a more coordinated approach or establishing connections with different electricity system data providers as backup sources for missing or incorrect data. For example, connecting directly to TSO platforms as a backup can overcome situations where those entities do not meet the deadlines to publish data on the Transparency Platform. The use of predicted power system carbon intensity data can represent an additional source of information for a hybrid methodology that combines both energy vulnerability and sustainability concerns. Collecting more data from consumers using the IR service could provide greater insights into the effectiveness of voluntary DR and how much flexibility these strategies can unlock from consumers. Linking recommendations with a dynamic pricing scheme would enable assessing the difference between voluntary and incentivized DR strategies.

In order to generalize the findings across the broader European context, future work should include a more diverse set of countries and consider larger sample sizes (i.e., a larger number of consumers). Although the pilots successfully demonstrate the implementation of the service, there is a need for extended pilots so that long-term data is available. This would allow us to analyze seasonal effects and possible degradation of consumer behavioral change over time, which could be an important factor for DR programs that do not include incentives.

Finally, the hierarchical approach for TSO-DSO cooperation in this work was solely centered on the task of system balancing by the TSO. Future work could include other tasks, such as congestion and voltage management, where the coordination between management at the national and utility scale is fundamental, and the same DR resources can be activated by multiple system operators.

## Resource availability

### Lead contact

Requests for further information and resources should be directed to and will be fulfilled by the lead contact, Carlos A. M. Silva (carlos.silva@inesctec.pt).

### Materials availability

The study did not generate new materials.

### Data and code availability

• This article analyzes existing, publicly available data. The categories of data, its source, and regulation article according to the ENTSO-E Transparency Platform are available in the key resources table.
• All original code associated with this document, namely code related to data acquisition, processing, and for instantiating and executing the Interoperable Recommender service, has been deposited at https://github.com/INESCTEC/interoperable-recommender-tso and is publicly available as of the date of publication. All original code associated with the DSO interface, the Energy App, the Semantic Interoperability Framework, and the *Wattchr* application has been deposited at https://gitlab.inesctec.pt/interconnect-public and is publicly available as of the date of publication. All original code associated with the AI4CS third party service has been deposited at https://github.com/local-ai-gr/AI4CS and is publicly available as of the date of publication.
• Any additional information required to reanalyze the data reported in this article is available from the lead contact upon request.

## Acknowledgments

The research leading to this work was carried out as a part of the InterConnect project (funded by European Union’s Horizon 2020, Grant agreement ID 857237). The sole responsibility for the content lies with the authors. It does not necessarily reflect the opinion of the Communications Networks, Content and Technology (CNECT) or the European Commission (EC). CNECT or the EC are not responsible for any use that may be made of the information contained therein. We want to thank Prof. Charalampos Skianis from the University of Aegean for his supervision and guidance to George Vlachodimitropoulos, who contributed to this research.

## Author contributions

Conceptualization: Carlos A. M. Silva, Ricardo J. Bessa, José R. Andrade, David E. Rua, and Fábio A. Coelho; methodology: Carlos A. M. Silva, Ricardo J. Bessa, José R. Andrade, Fábio A. Coelho, and David E. Rua; software: Carlos A. M. Silva, José R. Andrade, Fábio A. Coelho, Rafael B. Costa, and Ricardo J. Bessa; formal analysis: Carlos A. M. Silva, José R. Andrade, Fábio A. Coelho, Donatos Stavropoulos, and George Vlachodimitropoulos; resources: Donatos Stavropoulos, Spiros Chadoulos, Carlos Damas Silva, and George Vlachodimitropoulos; writing – original draft: Carlos A. M. Silva, Fábio A. Coelho, Donatos Stavropoulos, Spiros Chadoulos, Carlos Damas Silva, George Vlachodimitropoulos, and Ricardo J. Bessa; writing – review and editing: Carlos A. M. Silva, José R. Andrade, Fábio A. Coelho, David E. Rua, and Ricardo J. Bessa; visualization: Carlos A. M. Silva and José R. Andrade; supervision: David E. Rua and Ricardo J. Bessa; project administration: David E. Rua and Ricardo J. Bessa; funding acquisition: David E. Rua and Ricardo J. Bessa.

## Declaration of interests

The authors declare no competing interests.

## STAR★Methods

### Key resources table

**Table:** 

REAGENT or RESOURCE	SOURCE	IDENTIFIER
**Deposited data**		

Actual Total Load	ENTSO-E TP^51^	6.1.A
Day-ahead Total Load Forecast	ENTSO-E TP^51^	6.1.B
Day-ahead Aggregated Generation	ENTSO-E TP^51^	14.1.C
Day-ahead Generation Forecasts for Wind and Solar	ENTSO-E TP^51^	14.1.D
Actual Generation per Production Type	ENTSO-E TP^51^	16.1.B, 16.1.C
Installed generation capacity per unit	ENTSO-E TP^51^	14.1.B
Day-ahead Commercial Schedules	ENTSO-E TP^51^	12.1.F
Total Commercial Schedules	ENTSO-E TP^51^	12.1.F
Forecasted Day-ahead Transfer Capacities	ENTSO-E TP^51^	11.1
Forecasted Week-ahead Transfer Capacities	ENTSO-E TP^51^	11.1
Forecasted Month-ahead Transfer Capacities	ENTSO-E TP^51^	11.1
Forecasted Year-ahead Transfer Capacities	ENTSO-E TP^51^	11.1
Croatian pilot data	This paper	https://github.com/local-ai-gr/AI4CS

**Software and algorithms**		

Interoperable Recommender	This paper	https://github.com/INESCTEC/interoperable-recommender-tso
DSO Interface	This paper	https://gitlab.inesctec.pt/interconnect-public
Energy App	This paper	https://gitlab.inesctec.pt/interconnect-public
Semantic Interoperability Framework	This paper	https://gitlab.inesctec.pt/interconnect-public
Wattchr mobile application	This paper	https://gitlab.inesctec.pt/interconnect-public
Croatian pilot code	This paper	https://github.com/local-ai-gr/AI4CS

### Experimental model and study participant details

#### Pilot project participants

This study includes human participants that were enrolled in three pilot projects. The number of participants and other relevant aspects of the pilot projects are summarized in Table 2. More specifically, for the Portuguese, Greek, and Croatian pilot, recommendations were provided to a total sample size of 330 consumers, respectively 68, 150, and 112, during the pilot period.

Additional sociodemographic information was retrieved, although very limited and only in an aggregated manner for the set of consumers of each pilot, as further explained in the limitations of the study section, affecting the generalization of the results. We would like to highlight that this information was not used in the analysis due to its limited extent. Still, a summary with different levels of detail is included in each pilot project’s respective section. Some examples include the city of residence, professional and academic background, age and household composition.

Most of the participants became enrolled in the pilot projects by being employees of companies that participated in the pilots, trough referrals of friends and relatives of those employees, or by being customers of electricity supply companies that were part of the project.

Since all study participants received recommendations from the IR service during the pilot period, there is no formal definition of experimental groups that fit the analysis, as we do not have a treatment and control group. We do, however, have control days (i.e., days where the consumers did not receive recommendations), which are used in the analysis as historically comparable days to assess the impact of recommendations.

### Method details

This section exposes the methodology and the data sources behind the Interoperable Recommender (IR) service.

#### Interoperable recommender: data requirements

The Interoperable Recommender (IR) service depends on available data related to each country’s energy system operation. To support replicability, only publicly accessible data was used, namely data available in the ENTSO-E Transparency Platform.^51^ The data was requested via a Python client for the ENTSO-E API, using the *entsoe-py* package.^52^ A script is executed on a daily basis to collect the most recent data from the platform and store it in a local relational database. The first hour of execution was estimated by determining the hour at which most countries have the data (namely forecasts) available for the next day on the platform.

The requested data is detailed in the key resources table. It includes generation, load, scheduled commercial exchanges, and interconnection transfer capacity. Data can be considered as observed (which means data from the previous time periods or that it is known in advance) or forecasted (mostly day-ahead). Observed data (e.g., Actual Total Load and Actual Generation per Production Type) is mostly used as historical data to train forecasting models. Moreover, this data acquisition process includes some general information on the country’s power system, such as the installed capacity of each generation unit, so that the largest generation unit is known.

The retrieved data is initially resampled to an hourly resolution. A simple outlier detection method based on the Median Absolute Deviation (MAD) is then applied to identify abnormal values in the historical datasets. This process improves the forecasting accuracy of the ML-based regression models used to generate probabilistic forecasts. It is important to note that this service does not provide recommendations for countries where one or more variables (from the ENTSO-E TP) in the key resources table are missing (or considered a *NaN* value).

This study did not consider several European countries because they were not part of the InterConnect project. Still, different reasons exist for not being able to send recommendations for some European countries, the main one being missing data from the public source used (e.g., missing load and RES forecasts, historical load and RES data to train ML models, or import/export values for one or more interconnection).

#### Interoperable recommender: Service overview

**Figure 15:**
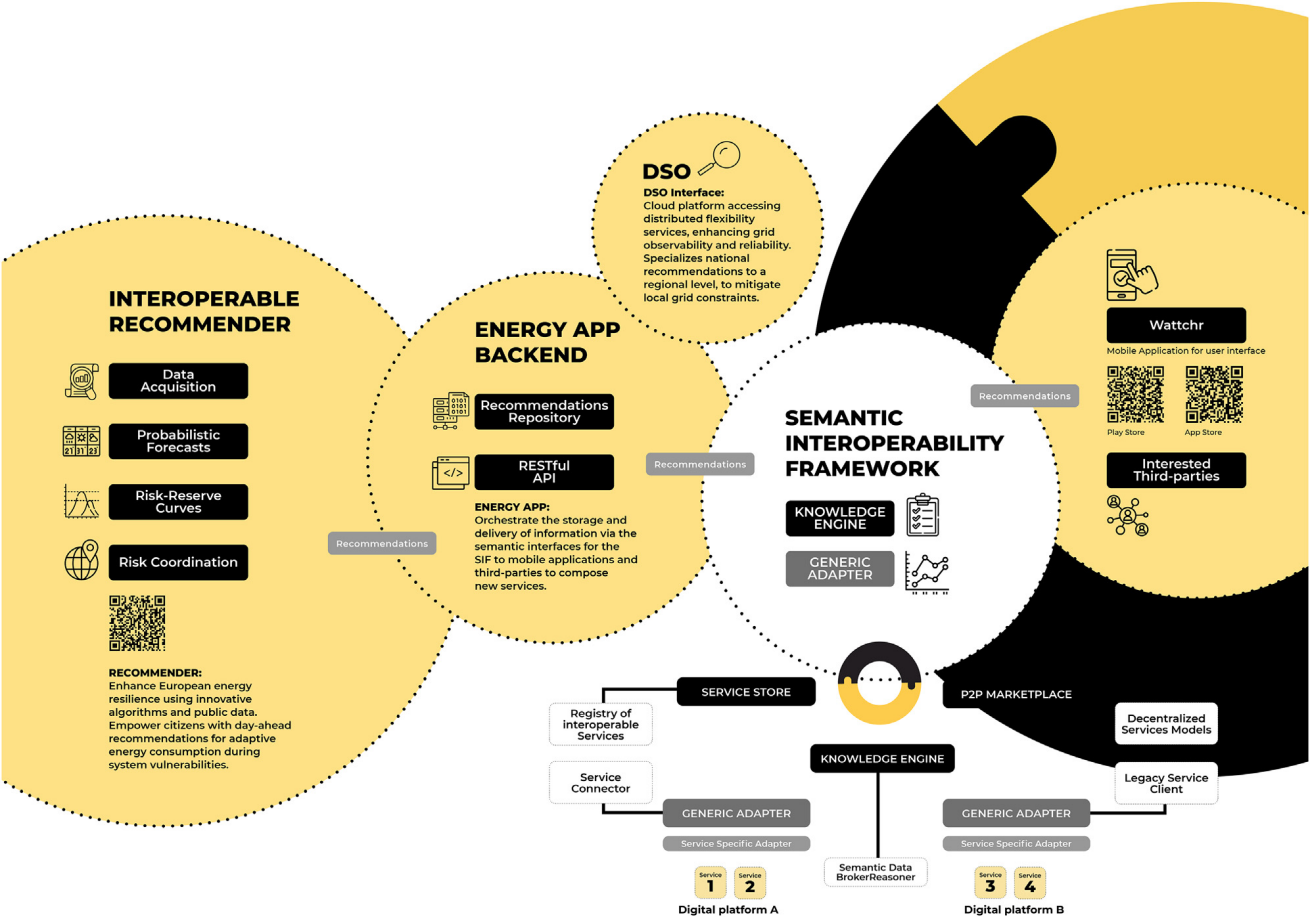
Schematic of interactions between IR, Energy App, DSOi, SIF, and third-party services The IR service and its supporting services. The IR delivers recommendations to the Energy App Backend. The DSOi can specialize in recommendations and update them on the backend. Third-party services that wish to use the recommendations use the Semantic Interoperability Framework, enabling seamless integration between the services.

Figure 15 depicts an overview schematic of the service, where the IR provides day-head country-level hourly recommendations to the backend of the Energy App via a representational state transfer (REST) application programming interface (API), which stores them in a relational database. The DSO can check and change the recommendations to a regional level, according to a certain specialization process conducted by the DSO Interface (DSOi). Third-party mobile applications that are interested in collecting the available recommendations (e.g., the pilot projects detailed in this work) can access them through the Semantic Interoperability Framework (SIF).

The IR service includes two main scripts: a) one for data acquisition, which accesses and processes the required data from the ENTSO-E Transparency Platform, and b) one for executing the service that calculates the risk and the appropriate recommendations. Due to data providers sometimes publishing data later than the expected deadlines in the ENTSO-E Transparency Platform, both scripts are executed sequentially, four times per day, to leverage the highest amount of data possible for all the countries. More precisely, the data acquisition pipeline is executed at 17h40, 18h40, 19h40, and 20h40 UTC. The recommendation calculation pipeline is executed at 18h30, 19h30, 20h30, and 21h30 UTC. If, during multiple executions, a recommendation for a specific country changes, an update is posted to the Energy App Backend.

The inputs to calculate the recommendations are provided by the data acquisition pipeline and are described in the previous section. The outputs of the service include, for each country, for each hour of the day, a data structure that includes the variables described in Table 4. More specifically, the output structure described includes the deterministic rule and calculated requirements for the reserve capacity, as well as the origin, direction, and magnitude of the recommendation.

**Table 4:** Outputs of the IR service

Variable	Type	Description
drr	float	Deterministic rule for reserve (DRR) in MW
reserve	float	Reserve capacity to meet risk threshold (R) in MW
origin	string	*individual* (recommendation to mitigate country risk) or *interconnection* (recommendation to help mitigate risk in neighboring country)
risk_evaluation	string	*not available* (no recommendation was issued), *healthy* (no risk of loss of load or generation curtailment), *increase* (country or neighbor at risk → increase energy consumption), or *decrease* (country or neighbor at risk → decrease energy consumption)
risk_level	int	Risk magnitude (0 - Healthy, 1 - Low, 2 - Medium, 3 - High, or 4 - Very high)

#### Probabilistic forecasting

A quantile-based probabilistic forecasting approach was applied for both RES and load day-ahead forecasts. The representation of uncertainty was defined with conditional quantiles. Intermediate quantiles, ranging from 5% to 95%, were computed using non-parametric methods, specifically linear quantile regression.^53^ The model relied on the following input features: a) a Fourier encoding of calendar variables such as hour of the day, weekday, and month, and b) deterministic forecasts (of load and RES) provided by ENTSO-E. Input Data was preprocessed by normalizing with a Standard Scaler^54^ (a transformation that removes the mean and scales features to unit variance). An isotonic regression model was used to ensure monotonicity, preventing intersections between quantile values predicted by individual regression models.

For the distribution’s tails (i.e., quantiles below 5% and above 95%), since data is typically sparse, non-parametric approaches are known to perform poorly.^55^ Therefore, we modeled the tails using a parametric approach, specifically with an exponential function, where the main parameter for each tail was estimated via maximum likelihood, as outlined in Gonçalves et al.^55^

To evaluate the quality of these quantile-based forecasts, we used the Continuous Rank Probability Score (CRPS, Equation 1),^56^ a metric that jointly quantifies both calibration and sharpness of the forecast. $L_{\tau }$ denotes the quantile loss function,^56^ $P_{t+k}$ represents the observed value of load or generation at horizon t+k, and ${\hat{P}}_{t+k|t}^{\left(\tau \right)}$ is the quantile (τ) forecast of load or generation, computed at *t* for horizon t+k.

Additionally, the Interquantile Range (IQR) is another relevant metric for evaluating the size of the forecast intervals, representing the range of values likely to contain the observed value. In this work, it was used to quantify the spread between the 5% and 95% quantiles, as shown in Equation 2, providing a direct measure of the uncertainty associated with the probabilistic forecasts.

(Equation 1) $$CRPS_{t+k|t}=2\int_{0}^{1}L_{\tau }\left(P_{t+k},{\hat{P}}_{t+k|t}^{\left(\tau \right)}\right)d\tau$$

(Equation 2) $$IQR_{t+k|t}={\hat{P}}_{t+k|t}^{\left(\tau =95\%\right)}-{\hat{P}}_{t+k|t}^{\left(\tau =5\%\right)}$$

#### System margin and reserve requirements

The TSO is responsible for setting the requirements for operating reserves for the next day, including both the frequency restoration reserve (FRR) and the replacement reserve (RR). Deterministic and probabilistic approaches are available in the literature to address this issue. As shown in Matos and Bessa,^10^ a probabilistic approach can be interesting to follow when a maximum acceptable risk level is known. The approach taken in this work was similar.

For each country, the conditional probability distribution of the RES generation is computed for each hour of the next day, including both wind and solar energy forecast uncertainty. The conditional probability distribution of the total load is also computed for each hour of the next day, accounting for its forecast uncertainty. The probability distribution of the system’s margin is calculated as the difference between the RES generation and the load probability distributions. This calculation can be performed via a convolution (Equation 3) using the fast Fourier transform, as further detailed in Matos and Bessa.^10^

(Equation 3) $$P_{c,t}^{M}=\left(G_{c,t}^{RES}-L_{c,t}=z\right)=\sum_{k=-\infty }^{\infty }P_{G_{c,t}^{RES}}\left(G_{c,t}^{RES}=z+k\right)\cdot P_{L_{c,t}}\left(L_{c,t}=k\right)$$

To account for the remaining components of a country’s energy balance, one can deterministically add to the probabilistic distribution of Equation 3 (shift to the left or right) the following variables: i) the scheduled conventional generation, ii) the total scheduled commercial exchanges (imports and exports), and iii) hydropower pumping. The result is a conditional probability distribution (Equation 4).

(Equation 4) $$P_{c,t}^{M^{*}}=P_{c,t}^{M}+G_{c,t}^{conv}+SCE_{c,t}^{imp}-SCE_{c,t}^{\exp}-L_{c,t}^{pump}$$

Different reserve levels can then be tested by shifting the distribution of the system margin in the amount of each reserve level to be assessed. For each reserve level, risk indices can be computed. More specifically, the cumulative distribution of the negative margin indicates the Loss of Load Probability (LOLP), and the cumulative distribution of the positive margin indicates the Probability of Curtailing Renewable Energy (PCRE). This relates to setting the country’s operating reserve requirements for upward and downward operating reserves. After testing multiple reserve levels, a curve with risk as a function of reserve requirements *R* (risk-reserve curve) can be developed to support decision-making (an example of risk-reserve curves is available in Figure 2).

Finally, the operating reserve requirements ($R^{up}$ and $R^{down}$) are defined by establishing a maximum acceptable risk level and intercepting the previously described curve with that risk level. A risk threshold of 0.1% was defined for the risk indices (LOLP and PCRE), which stems from the typical requirements of having less than 9 hours per year with a deficit in the reserves.^57^

#### Country-level risk calculation

The operating reserve requirements set by the probabilistic approach on the system margin are compared with a deterministic rule for the reserve (DRR). The DRR is useful since no country-level data regarding the reserves has been known beforehand.

The DRR includes both the automatic FRR (aFRR) and the combination of the manual FRR (mFRR) and the replacement reserve (RR), Equation 5. The aFRR (Equation 6) is estimated using the Empiric Noise Management Sizing Approach defined by ENTSO-E^57^ and is still used by several system operators today,^58^ where $L_{\max}$ is the maximum anticipated consumer load for the control area over the period considered, and the parameters *a* and *b* are defined empirically as 10 MW and 150 MW, respectively. The mFRR and RR (Equation 7) consider as a reference the loss of the biggest generation unit that is connected to the network, and two error terms are added, related to the mean absolute percentage error (MAPE) of the generation and load forecasts, respectively. These details are based on current considerations made by the Portuguese TSO (further detailed by the Portuguese Energy Regulator, *Entidade Reguladora dos Serviços Energéticos*^59^).

(Equation 5) $$DRR_{c,t}=DRR_{c,t}^{aFRR}+DRR_{c,t}^{mFRR+RR}$$

(Equation 6) $$DRR_{c,t}^{aFRR}=\sqrt{a\cdot L_{\max}+b^{2}}-b$$

(Equation 7) $$DRR_{c,t}^{mFRR+RR}=G_{c}^{largest}+\epsilon^{G}+\epsilon^{L}$$

Inspired by the well-being model for power systems from Billinton et al.,^42^ a framework that categorizes the adequacy of a system’s reserve capacity, the system is considered *healthy* when the operating reserve requirements are lower than the DRR. This can be checked for both upward and downward reserve requirements ($R^{up}\leq DRR^{up}$, and $R^{down}\leq DRR^{down}$). When the system deviates from *healthy*, one can interpret it as being *at risk* (i.e., the loss of at least one component specified by the deterministic rule would lead to some limit violation, as specified by the well-being model). Moreover, a System State (SS) can be established, with a scale defined by the amount that the DRR exceeds the reserve requirements (normalized by the country’s largest generator, $G_{c}^{largest}$), for example, through a rule-based system with empirical thresholds (Equations 8 and 9) that can be defined by a human operator or expert. The System State can be negative or positive, depending on the direction (upward or downward).

(Equation 8) $$\alpha_{c,t}=\frac{R_{c,t}-DRR_{c,t}}{G_{c}^{largest}}$$

(Equation 9) $$SS_{c,t}=\left\{ \begin{array}{cc} 0, & \alpha_{c,t}\leq 0\\ 1, & 0<\alpha_{c,t}\leq 0.2\\ 2, & 0.2<\alpha_{c,t}\leq 0.4\\ 3, & 0.4<\alpha_{c,t}\leq 0.8\\ 4, & \alpha_{c,t}>0.8 \end{array}\right.$$

#### Accounting for interconnections

It is assumed that a country can mitigate the risk that a neighboring country faces and that only countries with no individual risk can contribute towards risk mitigation. A simple rule-based system was developed for this purpose.

For each country *c at risk* (i.e., with a recommendation to increase or decrease consumption) at time *t*, we gather the set of neighboring countries with an active interconnection that is not *at risk* (i.e., are *healthy*) on the same period. The scheduled commercial exchanges at time *t* between country *c* and each neighbor *n* are recalculated, considering that we can add or subtract the total reserve deficit $\left(R_{c,t}-DRR_{c,t}\right)$ of country *c*, if that country was recommended to, respectively, decrease or increase its energy consumption (Equation 10).

It is checked if the recalculated scheduled commercial exchanges surpass the predicted net transfer capacity of that interconnection for period *t* when country *c* is importing from or exporting to neighbor *n* (Equation 11). If the constraint is fulfilled, then neighbor *n* can contribute to mitigate the individual risk of country *c*, by adopting the same action (increase or decrease consumption) for that period. For the sake of simplicity, a System State of 1 or -1 is attributed to neighbor *n*. Another approach would be to recalculate the risk indices (LOLP and PCRE) of each neighbor to set the new System State, which would be time-consuming and difficult to apply when multiple neighbors exist for the same country.

(Equation 10) $$SCE_{c,n,t}^{*}=SCE_{c,n,t}^{imp}-SCE_{c,n,t}^{\exp}\pm \left(R_{c,t}-DRR_{c,t}\right)$$

(Equation 11) $$\left\{ \begin{array}{cc} \left|SCE_{c,n,t}^{*}\right|\leq \left|NTC_{c,n,t}^{imp}\right|\text{,} & SCE_{c,n,t}^{*}>0\\ \left|SCE_{c,n,t}^{*}\right|\leq \left|NTC_{c,n,t}^{\exp}\right|\text{,} & SCE_{c,n,t}^{*}<0 \end{array}\right.$$

Finally, it is necessary to coordinate the actions of the different countries. When it is found that a country could help mitigate the individual risk of more than one of its neighbors, this could result in conflicting recommendations (of simultaneous increase and decrease actions). Therefore, a simplified approach is to sum all the mitigation actions for a certain country (which are -1 or 1), and if the total is positive, then the country’s action is to increase. Otherwise, the country’s recommended action is to decrease.

#### DSO interface and regional-level constraints

The DSOi was created to facilitate interoperable data exchange between the DSO and other market participants, including service providers and flexibility aggregators.^60^ The DSOi supports use cases involving data sharing, flexibility provisioning, and network observability. This framework is designed to improve electricity network stability by providing localized network information. The DSOi’s role is to supply energy-efficient applications with data regarding potential stress points in the distribution network at specific user locations, requiring the development of advanced backbone features and functionalities to enable the exchange of local network data with mobile applications. The DSOi uses Azure cloud services and includes multiple microservices for data storage and processing, event-driven functions, a graphical user interface, and REST APIs.

More specifically, the DSOi is also responsible for specializing national-level signals provided by the IR service to the regional level, considering the local usage and constraints of the distribution network.

On the network side, three essential datasets are required for the basic setup of the DSOi. These include data on the characterization of network assets such as primary and secondary substations, namely their capacities, hourly usage factors, and the mapping of secondary substations to their respective zip code ranges. For the Portuguese pilot, this information was sourced from a Customer Relationship Management (CRM) software and a Geographic Information System (GIS).

The DSOi analyzes the hourly recommendations for a specific country and assesses their applicability across all regions of the country. The DSOi may adjust these recommendations for specific locations with a rule-based system, and this adjustment can be performed by three distinct specialization methods, which have different priorities:

• *Automatic* (low priority): The DSOi analyzes the historical transformer capacity usage of the primary network elements, specifically of the Primary Substations (PS) and Secondary Substations (SS). If the transformer capacity usage is high (a threshold of 80% was set for this work), the DSOi disallows any recommendations that would increase the load. This evaluation enables the signals provided to not overload the network in specific periods where some of the network elements are already characterized as overloaded.
• *Semi-automatic* (medium priority): A Flexibility Management Module was designed to identify flexibility needs from the DSO medium and low-voltage networks using a network planning tool named DPLAN.^61^ DPLAN operates on a day-ahead basis, forecasting congestion issues in specific areas, calculating the necessary mitigation measures, and sending these flexibility needs to the DSOi platform. The DSOi then initiates processes to mobilize flexibility through flexibility service providers to secure explicit flexibility services. Given that these are real needs of the distribution system, they are crucial for specializing in IR-generated network signals. In this approach, rather than merely assessing whether a signal poses a risk to the local network, the DSO proactively aligns the signal with the identified flexibility needs for the targeted areas. This involves analyzing flexibility needs loaded into the DSOi Flexibility Management module and reflecting them in the region-level recommendations.
• *Manual* (high priority): The operator manually selects the desired hourly recommendation for the zip codes that relate to a specific network element (either PS or SS).

**Figure 16:**
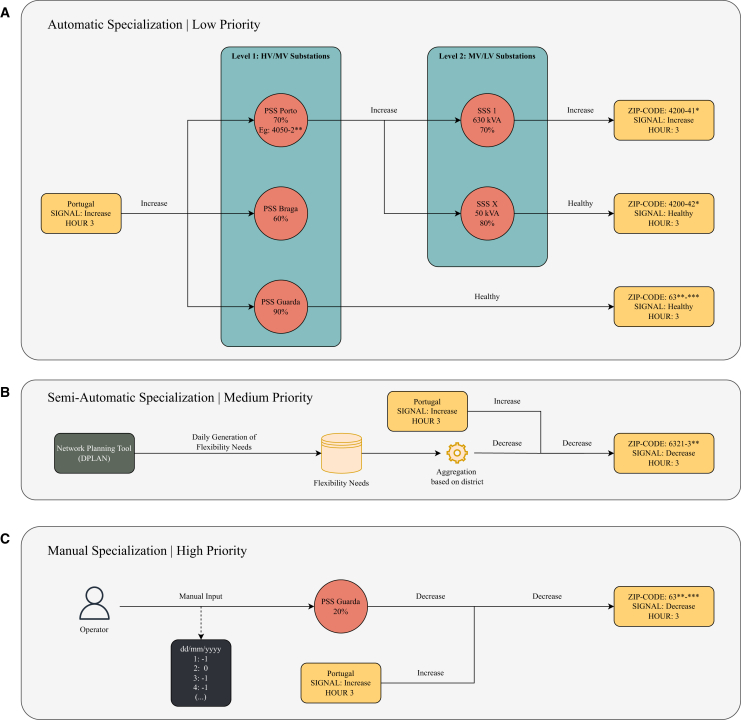
DSOi specialization methods The available specialization process of the recommendations deployed by the DSOi enables changing the recommendations for specific zip codes according to network conditions and other DSO needs. (A) The *Automatic* process is the lowest priority one and checks for overloaded primary and secondary substations to avoid recommendations to increase consumption in those periods. (B) The *Semi-automatic* process requests the flexibility needs from a network planning tool from the DSO that predicts congestion issues, requests flexibility, and adapts the recommendations accordingly. (C) The *Manual* process has the highest priority and requires an operator to submit specific recommendations for certain zip codes and periods.

The method is further illustrated in Figure 16. A recommendation to increase consumption was set for Portugal at 3 AM. In the *Automatic* specialization process, due to one primary substation (*PSS Guarda*) and one secondary substation (*SSS X*) being overloaded, the signal was changed for consumers to keep their usual consumption in certain zip codes associated with the substations’ near-by areas. In the *Semi-automatic* specialization process, for the same hour, the DPLAN tool had stored a request for flexibility to decrease consumption. Therefore, for the areas where flexibility was requested, the signal was changed for consumers to decrease their consumption based on their zip codes. Finally, for the *manual* specialization process, manual input was provided by an operator, who requested a decrease in consumption for the same hour. Therefore, the recommendation was changed so consumers could decrease their consumption instead of increasing it.

Following the necessary adjustments to the country-level recommendations, the DSOi sends the list of updated region-level recommendations to the Energy App backend.

#### Semantic interoperability framework

The cross-domain semantic interoperability is the transversal innovation of the InterConnect project, which is embodied through the Semantic Interoperability Framework (SIF).^24^ The SIF departs from a proven concept of distributed connectors or gateways, i.e., Service Adapters, but considers SAREF^25^ as ground to bring meaning to data in favor of considering a fixed data model as the interoperability agreement between stakeholders. Becoming semantically interoperable goes beyond the ability of systems to exchange information with correct syntax (syntactic interoperability) to the automatic and correct interpretation of the meaning of information. Ultimately, the SIF unlocks a semantically interoperable interface that allows stakeholders’ services to share and query knowledge represented according to SAREF instead of using syntactic interfaces with strict integration and querying options limited by the chosen data model. This means any question that fits the knowledge can interact with a component without relying on a matching API that was defined and implemented by the component.

The SIF bundles a toolset to enable interoperability up to the semantic level, namely: the Service Adapter as the connector adopted by digital platforms via a Service Specific Adapter (SSA); the distributed Semantic interoperability layer, handling knowledge exchange and querying between adapters; the Service Store providing a catalog of interoperable services, knowledge explorer and certification of services; the peer-to-peer marketplace for the instantiation of services devoted to fully distributed applications; and a set of tools to ensure security, privacy, and governance transversal capabilities.

The cornerstone of the SIF lies within its interoperability layer, which operates as a mediator and reasoner that orchestrates data exchange to fulfill question-answering and publish-subscribe requirements. This is based on a common language, i.e., ontology (SAREF), which defines the concepts and relationships in that domain, gearing a metadata model that steers data exchange and reaches seamless interoperability. To support question answering alongside publish-subscribe interactions, it provides two pairs of interactions: ASK/ANSWER and POST/REACT. The former pair enables question answering, and the latter enables publish-subscribe interactions. In the data exchange path, stakeholders link with these types of interactions depending on their role as data providers or consumers. These interactions are paired: ASK interactions are satisfied by ANSWER interactions, and POST interactions by REACT interactions. ASK is used to query knowledge from other knowledge bases, and ANSWER (its counterpart) is used to respond to such queries. POST is used to publish events or other messages, and REACT (its counterpart) is used to react to them.

Each stakeholder adopts an SSA holding a knowledge base that includes a registry of the configured knowledge interactions along with their metadata descriptions. When a request for knowledge is triggered, i.e., a knowledge interaction is activated, the holding knowledge base determines what metadata and outbound knowledge base the request should be forwarded. These capabilities are represented using *graph patterns* as depicted in Listing 1. This is a representation that is derived from *basic graph patterns* (https://www.w3.org/TR/sparql11-query/#BasicGraphPatterns) from the SPARQL language. Graph patterns consist of *triples* with a *subject*, *predicate*, and *object*.

Listing 1 **Recommender graph pattern considered for the ASK-ANSWER knowledge-interaction pairs**

?id dc:creator ?created_by .

?id ic-data:hasCreationTime ?created_at .

?created_at rdf:type time:instant .

?id gn:countryCode ?country_code .

?country_code rdf:type iso3166:Alpha2Code .

?id ic-data:hasEffectivePeriod ?period .

?period rdf:type time:interval .

?period time:hasBeginning ?sdt .

?sdt time:inXSDDateTimeStamp ?start_datetime .

?period time:hasEnd ?edt .

?edt time:inXSDDateTimeStamp ?end_datetime .

?id gn:postalCode ?zip_code .

?id ic-data:hasDataPoint ?data .

?data s4ener:hasValue ?risk_level .

?risk_level rdf:type s4ener:EventActionConsume .

?risk_level rdf:type xsd:integer .

?data s4ener:hasValue ?risk_evaluation .

?risk_evaluation rdf:type s4ener:EventActionConsume .

?data saref:hasTimestamp ?datetime .

?datetime rdf:type time:instant .

Knowledge exchange between knowledge bases is triggered by the proactive knowledge interactions: ASK and POST. When such a knowledge interaction is triggered, the interoperability layer finds the corresponding ANSWER or REACT interactions whose graph patterns match and mediates the data exchange by finding bindings for the variables in a graph pattern.

When triggering a proactive knowledge interaction, the knowledge base can provide initial bindings to the data exchange. This provides an easy way to filter the data exchange. For example, when a knowledge interaction involves several measurements of different sensors, the knowledge base that asks for sensor measurements can include a specific sensor in the binding set, which filters the results so that only measurements of that specific sensor are included.

The SIF acts as the key enabler within the CERF, providing a solid basis for implementing new and enhancing existing applications that face end consumers and provide them with actionable recommendations. The SIF allows third parties to access the recommendations with the full semantic context of their representation but also allows for specializations to be included back in the knowledge base of the recommender system. In this paper, the recommendations generated are disseminated in an interoperable way through the graph pattern in Listing 1. Prefixes are considered in Listing 2. This graph pattern is considered an ASK-ANSWER knowledge interaction. The use of this interface allows stakeholders to ask for knowledge matching this pattern, completely or partially, to acquire corresponding results. Finally, it allows for stakeholders collecting the ASK reply to have complete information on the specification and data representation formats for each field.

Listing 2 **Recommender graph pattern prefixes**

(gn,https://www.geonames.org/ontology#);

(s4ener,https://saref.etsi.org/saref4ener/);

(ic-data,http://ontology.tno.nl/interconnect/datapoint#);

(time,http://www.w3.org/2006/time#);

(saref,https://saref.etsi.org/corehasTime);

(rdf,http://www.w3.org/1999/02/22-rdf-syntax-ns#);

(iso3166,http://purl.org/dc/terms/ISO3166);

(xsd,http://www.w3.org/2001/XMLSchema#);

### Quantification and statistical analysis

There are no statistical tests included in this study. Basic statistical calculations were performed in Python. Some examples include: computed quantiles detailed in Figure 1; the calculation of forecasting skill metrics (MAE, IQR, and CRPS), included in Table 1, which were averaged for the 75 days of analysis, as explained in the Results; the first and third quartiles, and median, of reserve levels presented via a boxplot in Figure 3; the calculation of marginal histograms (per hour of day, and per country) in Figures 6 and 7; and the calculation of mean, maximum and minimum profiles of electricity consumption in Figures 11, 12, and 13 for Portugal (data for 25 consumers) and Greece (data for 33 consumers), considering 3 historically comparable days.
